# An artificial intelligence prediction model based on extracellular matrix proteins for the prognostic prediction and immunotherapeutic evaluation of ovarian serous adenocarcinoma

**DOI:** 10.3389/fmolb.2023.1200354

**Published:** 2023-06-14

**Authors:** Tianxiang Geng, Mengxue Zheng, Yongfeng Wang, Janne Elin Reseland, Athina Samara

**Affiliations:** ^1^ Department of Biomaterials, FUTURE, Center for Functional Tissue Reconstruction, Faculty of Dentistry, University of Oslo, Oslo, Norway; ^2^ Laboratory of Reproductive Biology, Faculty of Health and Medical Sciences, University of Copenhagen, Copenhagen, Denmark; ^3^ Department of Obstetrics and Gynecology, Seventh People’s Hospital of Shanghai University of Traditional Chinese Medicine, Shanghai, China

**Keywords:** extracellular matrix, ovarian serous adenocarcinoma, artificial intelligence, prognosis, immunity therapy

## Abstract

**Background:** Ovarian Serous Adenocarcinoma is a malignant tumor originating from epithelial cells and one of the most common causes of death from gynecological cancers. The objective of this study was to develop a prediction model based on extracellular matrix proteins, using artificial intelligence techniques. The model aimed to aid healthcare professionals to predict the overall survival of patients with ovarian cancer (OC) and determine the efficacy of immunotherapy.

**Methods:** The Cancer Genome Atlas Ovarian Cancer (TCGA-OV) data collection was used as the study dataset, whereas the TCGA-Pancancer dataset was used for validation. The prognostic importance of 1068 known extracellular matrix proteins for OC were determined by the Random Forest algorithm and the Lasso algorithm establishing the ECM risk score. Based on the gene expression data, the differences in mRNA abundance, tumour mutation burden (TMB) and tumour microenvironment (TME) between the high- and low-risk groups were assessed.

**Results:** Combining multiple artificial intelligence algorithms we were able to identify 15 key extracellular matrix genes, namely, *AMBN, CXCL11, PI3, CSPG5, TGFBI, TLL1, HMCN2, ESM1, IL12A, MMP17, CLEC5A, FREM2, ANGPTL4, PRSS1, FGF23*, and confirm the validity of this ECM risk score for overall survival prediction. Several other parameters were identified as independent prognostic factors for OC by multivariate COX analysis. The analysis showed that thyroglobulin (TG) targeted immunotherapy was more effective in the high ECM risk score group, while the low ECM risk score group was more sensitive to the RYR2 gene-related immunotherapy. Additionally, the patients with low ECM risk scores had higher immune checkpoint gene expression and immunophenoscore levels and responded better to immunotherapy.

**Conclusion:** The ECM risk score is an accurate tool to assess the patient’s sensitivity to immunotherapy and forecast OC prognosis.

## Introduction

Ovarian cancer (OC) is one of the most common gynaecological malignancies. According to the Global Cancer Observatory of the World Health organization (WHO) international agency for research on cancer, a total of 207,252 new fatalities due to ovarian cancer were reported in 2020, placing it 14th out of 36 different types of tissue cancers (World Health Organization International Agency for Research on Cancer, 2020). Most ovarian malignancies originate from epithelial cells, and the most prevalent histological subtype of epithelial ovarian cancer is ovarian serous adenocarcinoma (Heintz et al., 2006). Early OC detection is the best treatment scenario, but as OC presents with nonspecific symptoms and reflects detection, most patients are given a stage III diagnosis, indicating that the disease has spread throughout the peritoneum and/or has involved the lymph nodes (Prat and FIGO Committee on Gynecologic Oncology, 2014). A multi-stage evaluation is necessary to manage OC, to determine personalized treatment, and to predict the presence of distant metastases, tumour stage and prognosis.

As a new treatment option, immunosuppressants, address the tumour microenvironment (TME) (Pitt et al., 2016). For ovarian cancer, this cutting-edge therapeutical approach is recently being studied and applied (Yang et al., 2022, 2023). Despite the fact that many variables have been demonstrated to predict the therapeutic effectiveness of immunosuppressant’s, the accuracy of this strategy still needs to be improved (Gibney et al., 2016). Tumour development, spread and invasion are all dependent on the TME (Schreiber et al., 2011; Lei et al., 2020), which contains multiple cell types, including stroma, vasculature, secretory factors, surrounding stroma and the internal environment of the tumour cells. As the TME is primarily determined by the genomic landscape of the tumour, several algorithms have been developed to predict tumour purity and estimate the abundance of tumour-infiltrating immune cells based on gene expression profiles (Tamborero et al., 2018). These include CIBERSORT, MCP, Xcell, EPIC, ESTIMATE, Timer, IPS, and QuantiSeq.

As an essential component of TME, the non-cellular network surrounding the cells, known as the extracellular matrix (ECM), is tightly associated to the pathophysiology of healthy and cancerous tissue (Frantz et al., 2010; Henke et al., 2020; Zhu et al., 2022). This renders ECM a crucial study niche for the initiation, progression, dissemination, and furthermore treatment and prognosis of epithelial ovarian cancer (Ween et al., 2011). The metabolic disruption of various ECM protein-related factors derived from epithelial cells during tumorigenesis leads to the formation of a pro-tumorigenic microenvironment that favors tumor growth and metastasis. This is followed by tumour cell-mediated ECM remodelling, which ultimately promotes the survival of tumour cells at the expense of healthy tissue (Zigrino et al., 2005). Therefore, ECM proteins, which have bidirectional effects on the generation, recurrence and metastasis of tumour cells (Valmiki et al., 2021 should be considered key players to the treatment and prognosis of tumours (Donelan et al., 2022; Zhu et al., 2022).

An artificial intelligence algorithm, Random Forest (RF) has been recently employed to predict disease progression by virtue of its high performance and interpretability (Wu et al., 2021). A convincing predictive model can be constructed by combining analysis of gene expression data with diagnostic and therapeutic data. This model could be effective at forecasting patient survival, the course of the tumour, and recurrence following various types of treatment (Lin et al., 2022; Miao et al., 2022). Big data machine learning may also also be applied. Despite the recent advances in machine learning methods for ovarian cancer survival analysis, integration of multi-omics data with immunotherapy targeting is an approach that has not been thoroughly explored (Henderson et al., 2016; Belotti et al., 2022). This approach could be advantageous for the identification of potential therapeutic targets and may lead to improved outcomes for ovarian cancer patients.

In this study we used artificial intelligence algorithms to integrate multifaceted omics data with immunotherapy targets in ovarian cancer. Specifically, we employed the Random Forest and Lasso algorithms to process gene expression and survival data from the TCGA database. The tumour risk score was calculated to construct features for predicting OC prognosis and immunotherapy efficacy.

## Materials and methods

### Datasets and data quality control

Transcriptome expression profiles, somatic mutation data and clinical survival data were downloaded from the TCGA database (Supplementary Table S1). FPKM expression data from the UCSC XENA Project (https://xenabrowser.net/datapages/), which included the TCGA cancer gene expression sequencing data, were analysed together to increase the reliability of data analysis. Normal ovary tissue transcriptome sequencing data from the GTEx database (https://www.gtexportal.org/home/) were used as representative normal/healthy tissue data. We utilized the immune cell markers used in the Tumour MicroEnvironment (TME) analysis following the method described at Bindea et al. (2013) and ECM-related gene information was obtained from Naba et al. (2016). Following quality control measures on gene expression data and somatic mutations (SNPs and small INDELs), we filtered out 373 valid sample samples from the pool of 758 valid patient survival datasets of the TCGA-OV collection.

### Construction and evaluation of an ECM risk score model related to survival

The TCGA-OV data were randomly partitioned into a training set (*n* = 298) and a test set (*n* = 75). We used the randomForestSRC package (3.1.1) (Ishwaran et al., 2022) to down-size the 1068 ECM genes including survival information of OC patients. Further dimensionality reduction was performed by the Lasso algorithm in the glmnet package (4.1–2) (Friedman et al., 2010). Survival analysis of key genes in OV was performed with multivariate COX regression in the survival package (3.2–10) (Therneau, 2015).

### Differential expression and enrichment analyses

Two groups of patients with high and low-risk scores were generated. To calculate the differential gene expression between cancer data and normal tissue data we used DESeq2 v.1.36.0 (Love et al., 2014) in R (4.2.1). We performed Gene Ontology (GO) and Kyoto Encyclopedia of Genes and Genomes (KEGG) enrichment analyses using ClusterProfiler v.3.14.3 (Yu et al., 2012) in R (3.6.3). To find BP term enrichment, the Gene Set Enrichment Analysis (GSEA) (Subramanian et al., 2005) of ranked lists of differentially expressed genes was carried out. Significant enrichment in GSEA analysis is achieved when the False discovery rate (FDR) is 0.25 and an adjusted *p*-value of 0.05.

### Tumour microenvironment (TME) and somatic mutation analyses of the TCGA-OV dataset

We used the “maftools” package (2.12.0) (Mayakonda et al., 2018) for the calculation and evaluation of somatic mutations for each patient. The “drugInteractions” function was employed to analyse the correspondence between mutated genes and currently available genetic drugs based on the DGIdb database (Griffith et al., 2013). We further used multiple algorithms built into the IOBR package (0.99.9) (Zeng et al., 2021) to assess the immune cell infiltration level, including B cells, CD4^+^ T cells, CD8^+^ T cells, neutrophils, macrophages and dendritic cells. Then we explored the variations in immune infiltration and somatic mutation between groups with high and low-risk scores.

### Statistical analysis

The differences between the two datasets were determined using the Mann-Whitney *U* test (also known as the Wilcoxon rank sum test) and independent *t*-test. To evaluate between-group differences, one-way analysis of variance (ANOVA) with the Kruskal–Wallis test and chi-square test were utilised. Correlation analysis was conducted using non-parametric Spearman correlation tests. The connection between potential genes and overall survival was examined using a single-variable Cox regression analysis (OS). The difference was shown to be statistically significant when *p* < 0.05 was used (*p* < 0.05 *; *p* < 0.01 **; *p* < 0.001 ***).

## Results

### Screening and validation of ECM-related prognostic key genes

**FIGURE 1:**
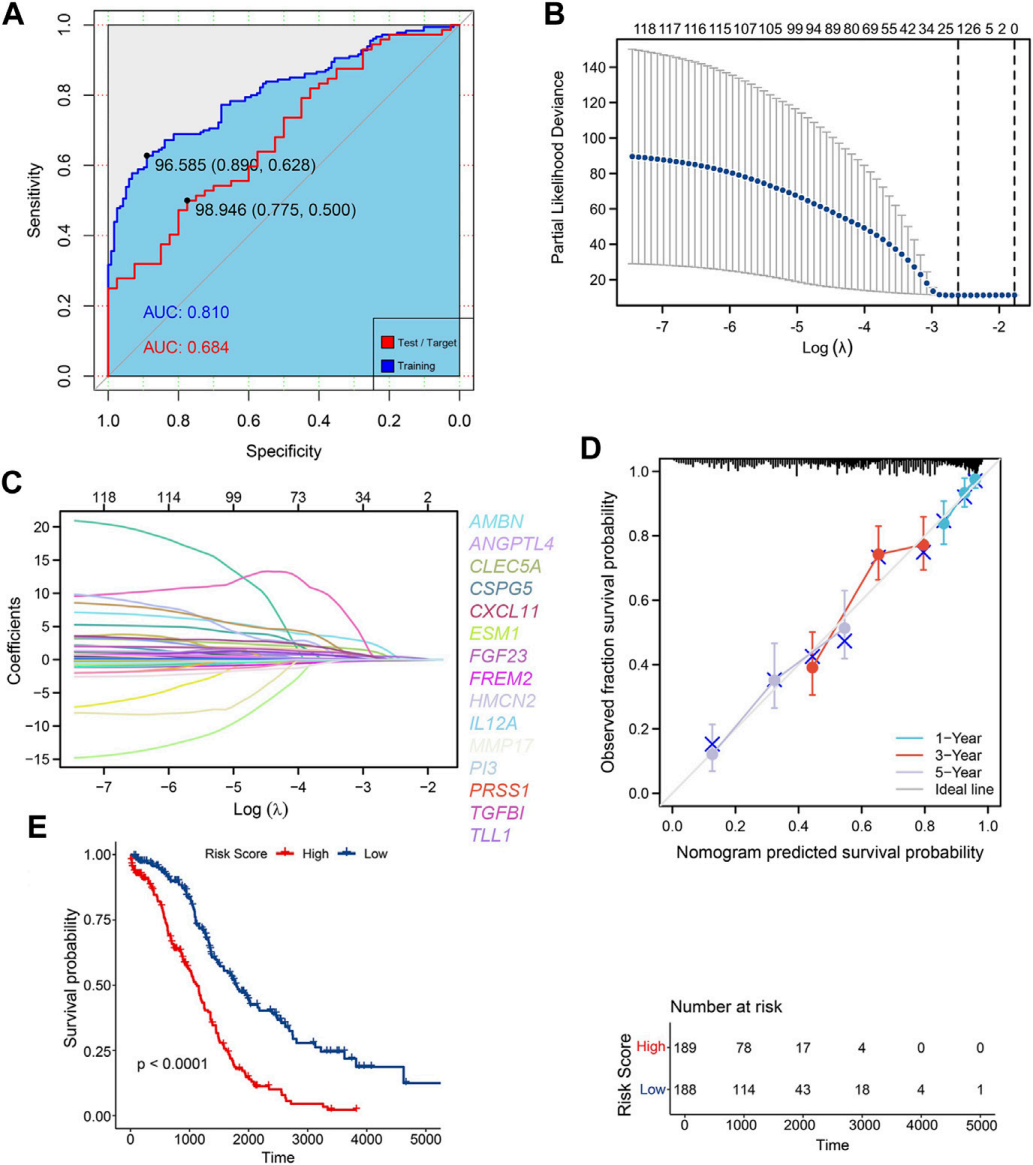
Construction of ECM risk score model **(A)** AUC for random forest train model (blue) and test model (red); **(B)** Lasso dimensionality reduction for random forest prognosis model; **(C)** Locus plot of all genes in random forest prognosis model; **(D)** Nomogram survival plot for 15 key prognosis genes; **(E)** KM survival plot for high/low ECM risk score group.

The clinicopathological characteristics of 379 OC patients in the TCGA database, are summarized in Table 1. The random forest algorithm was used to decrease the training set. 147 genes were screened out of 1068 ECM-related genes, and the accuracy of this survival prediction model was validated using the test set. The receiver operating characteristic curve (ROC) for the training set and test set were plotted separately, with the area under the curve (AUC) of 0.810 for the training set and 0.684 for the test set (Figure 1A).

**TABLE 1:** Characteristics of OC patients; source TCGA database.

Characteristics		N
Age	≤60	208
	>60	171
	Total	379
OS	Alive	147
	Dead	232
	Total	379
FIGO stage	Stage I	1
	Stage II	23
	Stage III	295
	Stage IV	57
	Total	376

The results of “lambda.min” of the Lasso algorithm were employed and 15 key genes closely related to prognosis were obtained (Figures 1B, C). These were Ameloblastin (AMBN), Chemokine (C-X-C motif) ligand 11 (*CXCL11*), Peptidase inhibitor 3 (*PI3*), Chondroitin sulfate proteoglycan 5 (*CSPG5*), Transforming growth factor (*TGFBI*), Tolloid-like 1 (*TLL1*), Endothelial cell-specific molecule 1 (*ESM1*), Matrix metallopeptidase 17 (*MMP17*), Angiopoietin-like 4 (*ANGPTL4*), Fibroblast growth factor 23 (*FGF23*), Hemicentin 2 (*HMCN2*), Interleukin 12A (*IL12A*), C-type lectin domain family 5, member A (*CLEC5A*), FRAS1 related extracellular matrix protein 2 (*FREM2*), serine protease 1 (*PRSS1*), and the gen prediction model with the risk coefficient of 15 genes given by the Lasso algorithm was constructed:

$$\begin{array}{ll} Risk\;score & =TGFBI\;*\;\left(0.0092\right)+CSPG5\;*\;\left(-0.0307\right)+PI3\;*\;\left(0.0481\right)+CXCL11\;*\;\left(-0.1219\right)+MMP17\;*\;\left(0.0512\right)\\ & +IL12A\;*\;\left(0.0084\right)+ESM1\;*\;\left(-0.0317\right)+HMCN2\;*\;\left(0.0707\right)+TLL1\;*\;\left(0.4759\right)+FGF23\;*\;\left(0.0328\right)\\ & +PRSS1\;*\;\left(-0.0277\right)+ANGPTL4\;*\;\left(0.0245\right)+FREM2\;*\;\left(-0.0014\right)+CLEC5A\;*\;\left(0.0311\right)+AMBN\;*\;\left(1.2734\right) \end{array}$$

The Cox model was used to verify the predictive ability of the 15 key genes for the 1-year, 3-year and 5-year overall survival (OS), and the key genes fit well with the ideal line at the three-time points (Figure 1D). The TCGA-OV sample was divided into two groups with high and low-risk scores based on the average risk score. Furthermore, the Kaplan Meier (KM) curves of high/low-risk score groups were plotted, showing a significant difference between the high and low-risk score groups (Figure 1E). Additional multifactorial Cox models were used to analyse the relationship between the 15 key genes and ovarian cancer OS (Table 2), and we found that *AMBN, CXCL11, CLEC5A,* CSPG5 *FREM2, MMP17,* and *PI3* were independent prognostic factors for ovarian cancer.

**TABLE 2:** Multifactorial Cox survival analysis of the 15 key genes in TCGA-OV patients.

Characteristics	High	Low (Reference)	Univariate analysis		Multivariate analysis	
			Hazard ratio (95% CI)	*p* value	Hazard ratio (95% CI)	*p* value
TGFBI	186	187	1.118 (0.863–1.448)	0.398	0.822 (0.587–1.150)	0.252
CSPG5	186	187	0.754 (0.582–0.978)	0.033	0.811 (0.611–1.077)	**0.047**
P13	186	187	1.470 (1.133–1.908)	**0.004**	1.379 (1.044–1.820)	**0.023**
CXCL11	186	187	0.614 (0.472–0.798)	**<0.001**	0.567 (0.425–0.756)	**<0.001**
MMP17	186	187	1.689 (1.301–2.192)	**<0.001**	1.553 (1.150–2.098)	**0.004**
IL12A	186	187	1.010 (0.780–1.308)	0.939	0.942 (0.716–1.238)	0.666
ESM1	186	187	0.936 (0.723–1.213)	0.618	1.035 (0.783–1.369)	0.809
HMCN2	186	187	1.503 (1.157–1.953)	**0.002**	1.172 (0.871–1.578)	0.294
TLL1	186	187	1.400 (1.080–1.815)	**0.011**	1.285 (0.966–1.711)	0.085
FGF23	186	187	1.191 (0.919–1.543)	0.185	1.085 (0.821–1.436)	0.565
PRSS1	186	187	0.818 (0.630–1.061)	0.131	0.979 (0.738–1.299)	0.885
ANGPTL4	186	187	1.377 (1.061–1.786)	0.016	1.064 (0.794–1.424)	0.679
FREM2	186	187	0.724 (0.558–0.940)	0.015	0.633 (0.473–0.847)	**0.002**
CLEC5A	186	187	1.577 (1.213–2.051)	**<0.001**	1.444 (1.044–1.998)	**0.026**
AMBN			6.630 (2.508–17.526)	**<0.001**	8.544 (3.045–23.976)	**<0.001**

* Total number of patients 373.

Statistically significant values are indicated in bold.

### Differential expression analysis and functional enrichment of high and low ECM risk score groups

**FIGURE 2:**
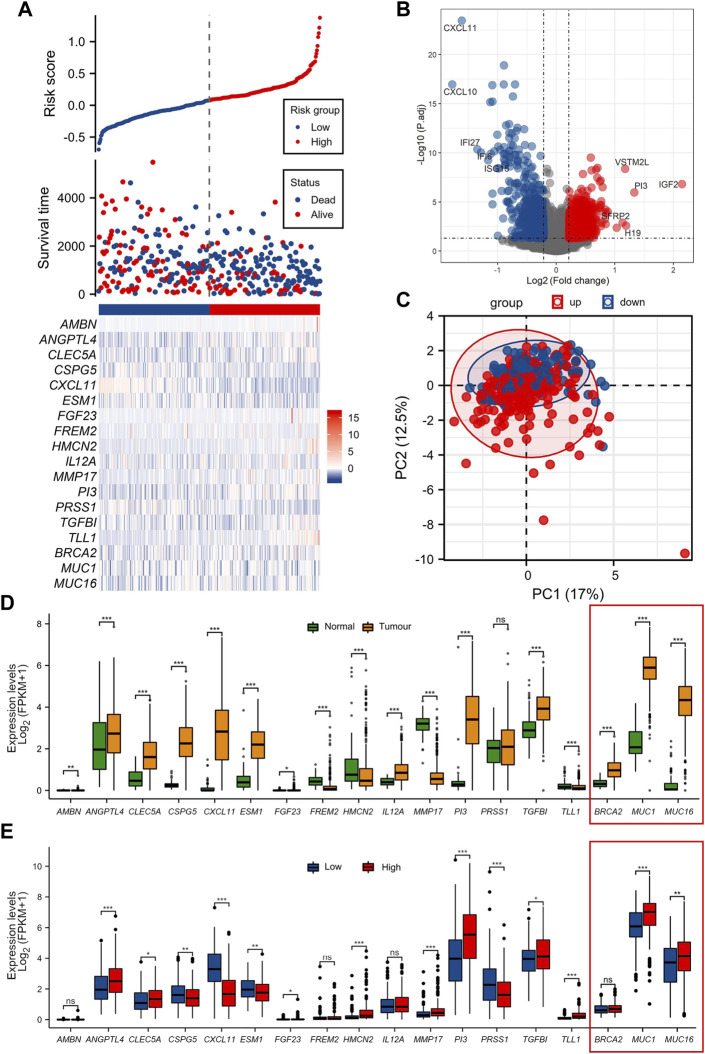
Differential analysis of function between high/low ECM risk score group **(A)** Information of sample group based on ECM risk score and 15 key prognosis gene expression heatmap. **(B)** Volcano map of differential gene expression analysis in TCGA-OV. **(C)** Principal component analysis (PCA cluster) based on the 15 key prognostic genes in TCGA-OV. **(D)** 15 key genes expressed in normal ovary and TCGA-OV. **(E)** 15 key genes expression in ECM high/low-risk score groups. The genes *BCRA2*, *MUC1* and *MUC16* (in red frames) have been functionally characterized in other studies, for their prognostic role in OC, and used as a reference.

The ECM risk score, survival information, and one-to-one correspondence to the expression of the 15 key genes for each sample in TCGA-OV are presented in Figure 2A. The results of differential gene expression of high vs. the low-risk score group showed that 1004 genes were significantly upregulated (logFC > 0.4, adj. *p* < 0.05), and 378 genes were significantly downregulated (Figure 2B).

We documented that there is a substantial difference in the extracellular matrix-related processes in the enrichment of GOKEGG functions (Supplementary Figure S1A). Additionally, it was shown that biological processes associated with immune cells differed dramatically (GO:0071621, GO:0043030 et al.). Two immune-related pathways were revealed to be blocked in the high ECM risk score group in the GSEA results (Supplementary Figure S1C). Based on the 15 key prognostic genes in the TCGA-OV at the principal component analysis (PCA cluster), there was little difference between the high and low-risk subgroups in the PC1 and PC2 dimensions (Figure 2C).

We also examined the expression of the 15 key genes in normal ovarian tissue vs. the TCGA-OV collection, and in low/high ECM risk score groups (Figures 2D, E). In the expression analysis of normal vs. tumour tissues, only *PRSS1* was not significantly differentially expressed in normal versus tumour tissues. The expression levels of *TLL1, HMCN2, FREM2 and MMP17* were significantly higher in normal ovarian tissues than in tumour tissues. *AMBN, TGFBI, CSPG5, PI3, CXCL11, ESM1, FGF23, ANGPTL4, CLEC5A* and *IL12A* all showed significantly higher expression in tumour tissues samples.

In the differential expression analysis of low/high ECM risk score groups, *AMBN, IL12A and FREM2* were not statistically different, whereas *PI3, TGFBI, TLL1, HMCN2, MMP17, CLEC5A, ANGPTL4, FGF23* had higher expression in the high-risk group and *CXCL11, CSPG5, ESM1, PRSS1* were highly expressed in the low-risk group.

The genes *BCRA2*, *MUC1* and *MUC16* (in the red-framed rectangles in Figures 1C, D, and the heat map), were also assessed to supplement our analysis with three genes from the same dataset, previously functionally characterized for their prognostic role in OC (Wang et al., 2007; Zhai et al., 2020; Custódio et al., 2022). The genes are also.

### Assessing the role of ECM risk score in tumour immune cell infiltration and immunotherapy response

**FIGURE 3:**
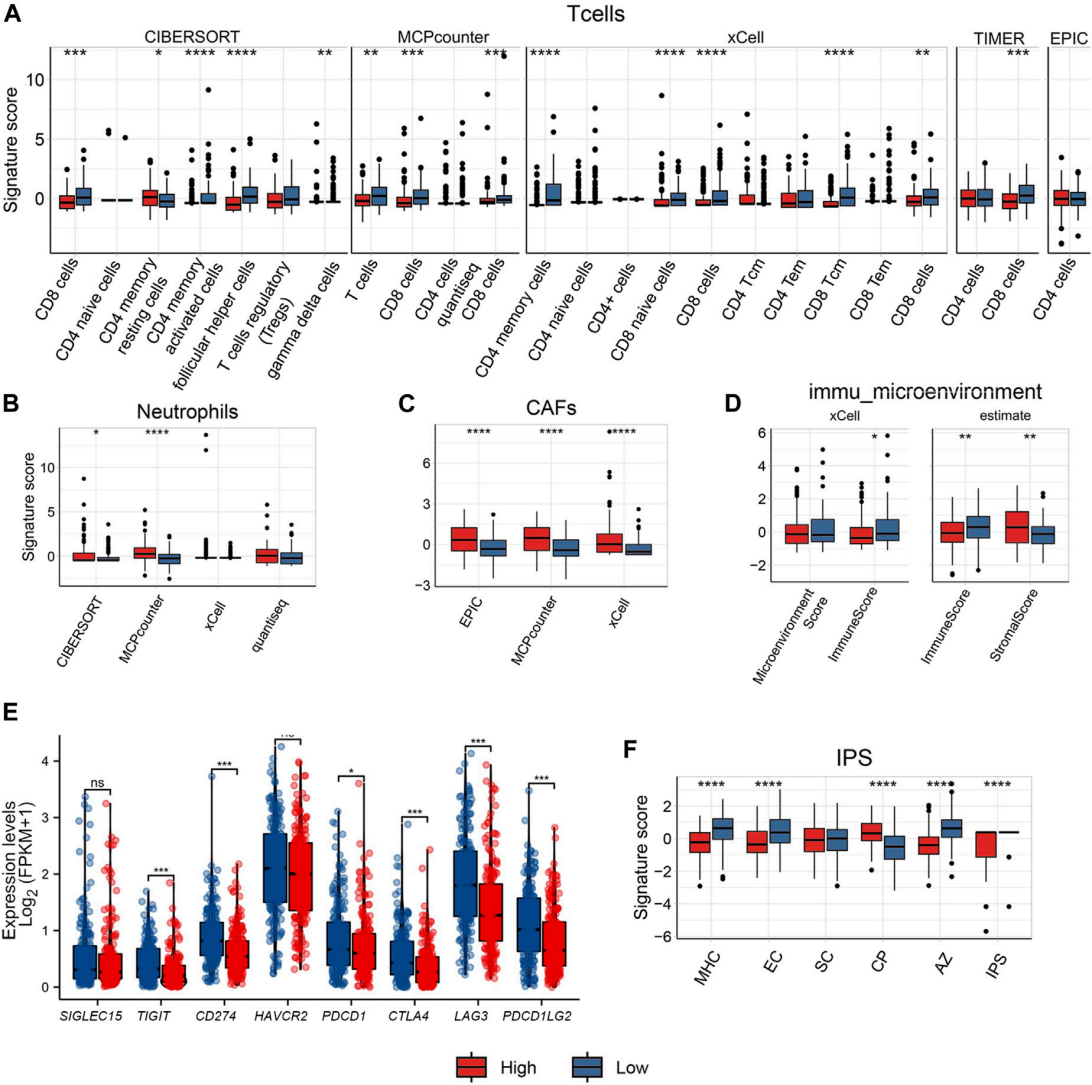
Comparison of immune cell infiltration between high/low ECM risk score group groups in TCGA-OV. Comparison with several algorithms for T cell **(A)** infiltration**,** Neutrophil **(B),** cancer-associated fibroblast (CAFs) **(C)**, Immune microenvironment score **(D)** infiltration, expression levels of 8 immune checkpoint genes in high/low ECM risk score group in TCGA-OV **(E)**, and IPS score in high/low ECM risk score group in TCGA-OV **(F)**.

The analysis using almost all algorithms, documented that CD8 T cells showed a significant difference, with lower levels of infiltration in the high-risk group than in the low-risk group. In the high-risk group, the signature score of CD4 T memory resting cells was higher, and lower in all other T cells (Figure 3A). Neutrophils scored variable results among the four algorithms: there were group differences in the infiltration levels of neutrophils in the CIBERSORT and MCPcounter algorithms, with both algorithms showing higher levels in the high-risk group (Figure 3B). According to the EPIC, MCPcounter, and xCell algorithms, the high-risk group had higher numbers of cancer-associated fibroblasts (CAFs) (Figure 3C).

Both the xCell and ESTIMATE algorithms indicated a lower immune microenvironment score for the high-risk group when computing the immune microenvironment score. The high-risk group displayed a higher stromal score in the ESTIMATE algorithm, indicating the presence of more stromal cells (Figure 3D).

The analysis of B cells also showed high variation among the algorithms used: significant differences between groups were only documented by the xCell algorithm; naive B cells and plasma cells showed group differences under the CIBERSORT algorithm but not when the xCell algorithm was employed (Supplementary Figure S2). There was no discernible difference between the two groups in monocytes (Supplementary Figure S2). Only xCell revealed group differences in DC cells (Supplementary Figure S2). NK cells only showed between-group differences under the *MCPcounter* and *quantiseq* algorithms, but there was an opposite trend: NK cells showed relatively low levels in the high-risk group under the *MCPcounter* algorithm but relatively high levels in the high-risk group under the quantiseq algorithm (Supplementary Figure S2). The high-risk group’s Macrophage M1 levels were only marginally different to the low-risk group according to the CIBERSORT and xCell results, and there was no difference between groups when the EPIC method was used (Supplementary Figure S2).

We also extracted the expression levels of eight immunological checkpoint genes (Figure 3E). The expression levels of six immune checkpoint genes (*TIGIT, CD274, PDCD1, CTLA4, LAG3, and PDCD1LG2*) was higher in the low-risk group than in the high-risk group, with the exception of *SIGLEC15* and *HAVCR2*. The IPS score for the major histocompatibility complex (MHC), and for senescent cells (SC) was greater in the low-risk group than in the high-risk group. Endothelial cells (EC) IPS score did not differ statistically significantly between the two groups. However, the high-risk group had a higher Classical Pathway (CP) IPS score than the low-risk group. Both the aggregated z-score (AZ) and the weighted total IPS showed that the low-risk group was higher than the high-risk group (Figure 3F).

### Validation of the prognostic function of ECM risk score in the TCGA-pan-cancer dataset

**FIGURE 4:**
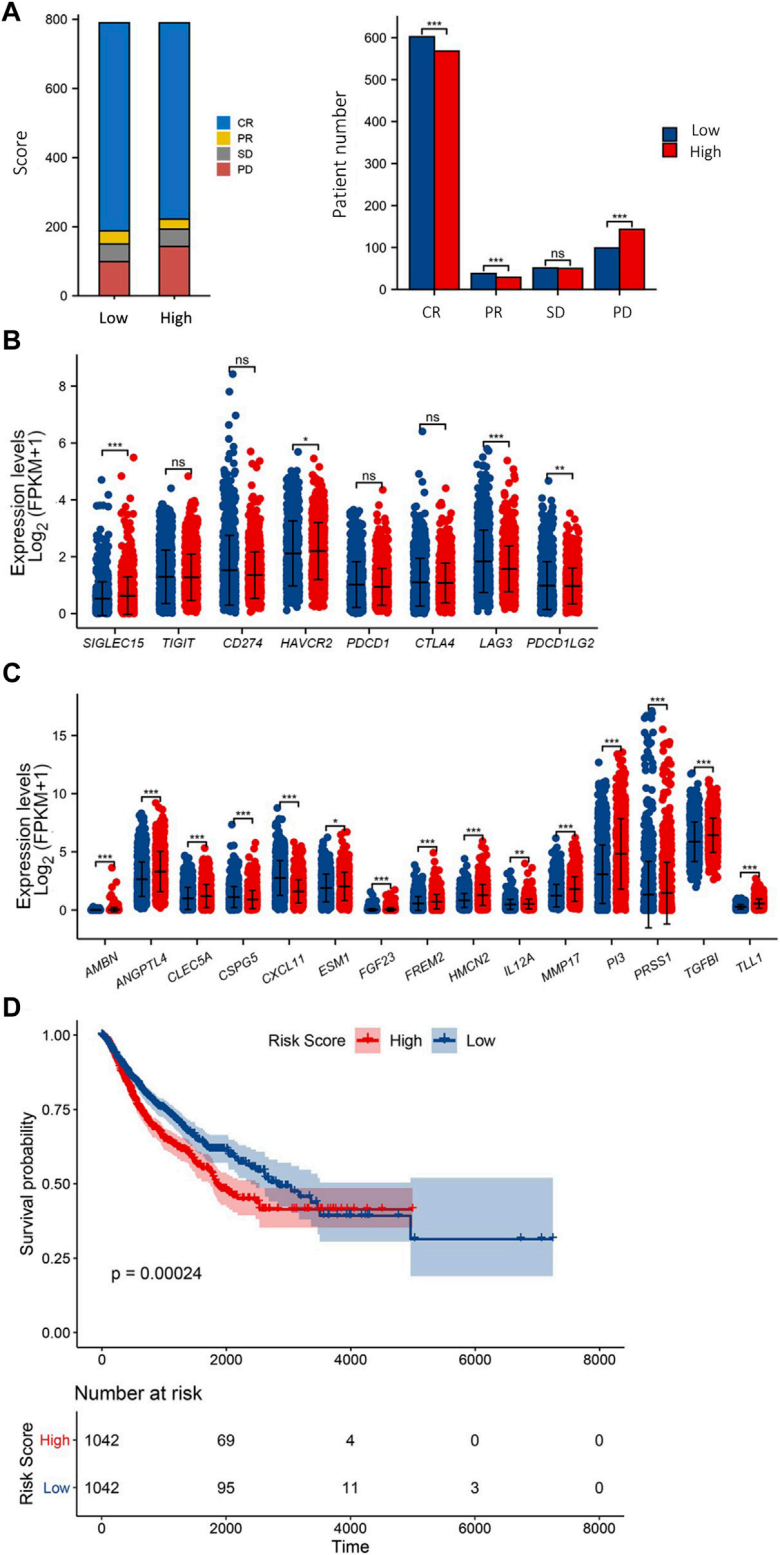
Validation of ECM risk key genes in TCGA Adenocarcinoma. First-course treatment outcome of high/low ECM risk score group in TCGA-Adenocarcinoma **(A)**. Expression levels of 8 immune checkpoint genes **(B)** and 15 ECM key genes **(C)** in high/low ECM risk score group in TCGA-Adenocarcinoma. KM survival plot for high/low ECM risk score group in TCGA-Adenocarcinoma **(D)**.

TCGA pan-cancer data with survival information were used to validate the ECM risk score. A total of 9162 “Primary Solid Tumour” data with both gene expression data and survival data were included in the analysis. We screened all adenocarcinoma expression data and survival data as a validation dataset. 2084 samples meeting the criteria were extracted, of which 1580 samples carried information on initial treatment outcome. We found that the low-risk group had a higher initial treatment Complete Response (CR) and Partial Response (PR), there was no significant difference in the number of patients with Progressive Disease (PD) between the low- and high-risk groups, and the number of patients with Stable Disease (SD) was significantly higher in the high-risk group than in the low-risk group (Figure 4A). We also analysed the expression levels of eight immune checkpoint genes in the high/low ECM risk score group (Figure 4B). Only four immune checkpoint genes showed significant differences between the two groups. Fifteen ECM’ key genes were extracted from all gene expression data, and the ECM risk score was calculated for each patient. The expression levels of 15 ECM key genes were differentially expressed in both the high and low-risk groups (Figure 4C). Computes the predicted survivor function for a Cox proportional hazards model and plots the KM curve (Figure 4D). The ECM risk score was found to be a good predictive tool for overall survival prognosis in the adenocarcinoma data. However, it was not adequate in effectively predicting for OS between the high and low-risk groups after 4000 days.

### Relationship between ECM risk score and tumour mutation burden (TMB)

We further investigated the connection between the ECM risk score and TMB because OC is marked by a high degree of somatic mutation. Missense Mutation was the main component of somatic mutation in the high/low ECM risk score group. In the single nucleotide variant (SNV) analysis showed that the highest rate of C > T was observed in both groups. Variants per sample of the high ECM risk score group were lower than in the low ECM risk score group (Supplementary Figures S3A, B). In both high/low ECM risk score groups, *CSMD3, TTN, TP53, FLG2, MUC16, FLG* and *FAT3* were found in the top 20 mutated genes (Supplementary Figures S3C, D). In comparison to the high-risk group, the mutation rates in *TP53, TTN* and *RYR2* were higher in the low-risk group, while those of *CSMD3, USH2A* and *FLG2* were nearly identical (Supplementary Figures S3E, F).

**FIGURE 5:**
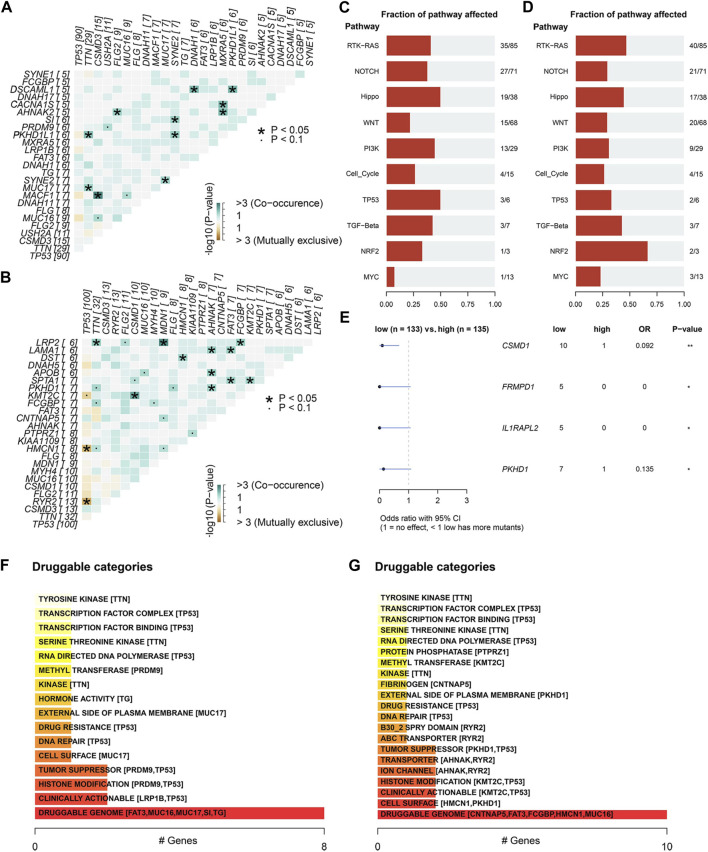
Differential analysis of somatic mutations between high/low ECM risk score group in TCGA-OV. The relation of the top 25 mutated genes in the high **(A)** and low **(B)** ECM risk score groups. Analysis of cancer-related key pathway components affected by somatic gene mutations in the high **(C)** and low **(D)** ECM risk score groups. Differential analysis of somatic gene mutatios between high/low ECM risk score group **(E)**. Oncogenic signalling pathways enrichment analysis in high **(F)** and low **(G)** ECM risk score group.

In the mutation Exclusive/Co-occurring analysis of the top 20 mutation genes, we found 11 pairs of genes with Co-occurring relationships in each of the high/low ECM risk score groups but 2 pairs of Exclusive in the low ECM risk score group (Figures 5A, B). We also examined the mutation status of the elements of eight signalling pathways that have been demonstrated to be crucial in the development of tumours (Sanchez-Vega et al., 2018). We found that the number of genes affected by somatic mutations in most signalling pathways was approximately the same between the two groups, except for *RTK-RAS, NOTCH, WNT,* and *PIK2* (Figures 5C, D). The expression levels of all somatic mutations were analysed between the high/low ECM risk score groups, and we found statistically significant differences in *CSMD1, FRMPD1, IL1PARL2* and *PKHD1*. All four genes showed a higher mutation rate in the low ECM risk score group (Figures 5D, E). Based on the differences in somatic mutations between the two groups, we enriched the analysis for drug-gene interactions, and the “Druggable Genome: was found to be highly enriched within both groups (Figure 5F, G).

Additionally, we documented that the low ECM risk score group was more responsive to RYR2 gene-related immunotherapy, whereas the high ECMs risk score group may be more responsive to TG-related immunotherapeutics (Supplementary Table S2). We also analysed the mutations in 15 key genes (Supplementary Figure S4).

## Discussion

Ovarian cancer is a malignant tumour with a high mortality rate. Because of its insidious onset, it is usually late-stage when obvious clinical symptoms appear (Prat J and FIGO Committee on Gynecologic Oncology, 2014). Patient prognosis is thus based on an accurate and reliable assessment. As the extracellular matrix is closely linked to epithelial cells, we aimed to construct a prognostic prediction model based on extracellular matrix proteins to evaluate the prognostic survival of patients with ovarian serous adenocarcinomas. We successfully filtered 15 key genes out of 1068 extracellular matrix-associated proteins, with excellent predictive ability on ovarian serous adenocarcinoma prognosis using the Random Forest and the Lasso algorithms.

We used the random forest algorithm as is a popular machine learning technique with documented outstanding performance in a wide range of predictive modeling tasks, including cancer prognosis prediction (Toth et al., 2019; Li Y et al., 2020). When analyzing tumor sequencing data, the relationship between variables and outcomes can be complex due to the volume of data. Random forest can handle a large number of input variables without overfitting, which has been a challenge for traditional models such as logistic regression or decision trees (Maroco et al., 2011; Lan et al., 2020). Additionally, traditional linear models are unable to handle the nonlinear relationship between a large amount of sequencing data and survival outcomes, but random forest can address this issue (Lee and Lim, 2019). Compared to support vector machines (SVM), random forest is less sensitive to outliers and missing data, which is important in cancer prognosis prediction, where data quality may vary and missing data is common (Pelckmans et al., 2005; Lee and Lim, 2019).

Among the 15 key genes generated by our our analysis, *CSPG5, CXCL11* and *ESM1* mRNAs were abundantly expressed in OC tissue compared to normal tissue. Cancer cells, fibroblasts, endothelial cells, and immune cells such as leukocytes, monocytes, and dendritic cells are primarily responsible for *CXCL11* production (Gao and Zhang, 2021) *CXCL11* is an effector chemokine regulating T cell recruitment that promotes effector immune cells (e.g., CD8T cells, Th1 cells, TH17 cells and antigen-presenting cells). Studies have shown that induction of *CXCL10* and *CXCL11* expression in breast cancer cells enhances the infiltration of CD8 T cells (Liu et al., 2011; Gao et al., 2019). In addition to its influence on tumour progression through its angiostatic effects (Strieter et al., 2006), CXCL11 is part of the CXCR7/CXCL11 axis that was shown to induce the epithelial–mesenchymal transition and metastatic behaviour of OC cells under ERα control (Benhadjeba et al., 2018). *ESM1* is a soluble proteoglycan expressed by vascular endothelial cells and is associated with inflammatory cell recruitment (Hung et al., 2020). Vascular endothelial dysfunction can be brought on by high levels of ESM1 (Kalantaridou et al., 2006; Rocha et al., 2014; Sun et al., 2019), whereas it has also been shown to be closely correlated with OC development and progression (Li et al., 2023). Our analysis showed that the tumour tissue had significantly higher *ESM1* expression levels than normal ovarian tissue, which may be associated with abnormally elevated cell proliferation and tumour tissue revascularization. Furthermore, CSPG5, also known as Neuroglycan C (NGC), is a protein originally associated with extracellular matrix production in the nervous system (Pintér et al., 2020) and shown to decrease first and then increase following ischemic and hypoxic injury, presumably associated with ECM damage repair (Matsui et al., 2005). Of note, two recent studies showed that expression of CSPG5 was significantly correlated with the prognosis of patients with epithelial OC (Su et al., 2021; Wang et al., 2023). In our study, higher *CSPG5, CXCL11* and *ESM1* expression and inflammatory cell infiltration, especially of CD8 T cells, were present in the low-risk group. The high level of immune cell environment may explain the better prognosis for overall survival in the low-risk group.

Interestingly, two proteins commonly associated with bone biology and development were identified among the ECM prognostic markers. AMBN was discovered as a tooth enamel matrix protein, playing an important role in enamel, cranial and long bone development. It was however demonstrated that AMBN was among four factors that were potential independent prognostic factors for prostate cancer (Xu et al., 2018). In our multifactorial Cox analysis, AMBN was shown to be one of the independent prognostic factors for ovarian serous adenocarcinomas. Furthermore, the analysis revealed a prognostic role for *FGF23*, the bone-derived hormone secreted by osteoblasts and osteocytes (Guo and Yuan, 2015). Previous studies have shown its expression alterations in breast cancer (Cekin et al., 2020) and identified that serum or plasma FGF23 concentrations are elevated in patients with advanced stage epithellal ovarian cancer (Tebben et al., 2005). According to our data, FGF23 was expressed in the tumours, and its expression levels were higher in the high-risk group.

*MMP17, PI3, TLL1, ANGPTL4,* and *TGFBI* have all been previously associated with cancer. Our analysis found that transcripts of all these five genes were expressed at higher levels in the high-risk group than in the low-risk group. *MMP17* has been associated with the maintenance of normal physiological function in vascular smooth muscle (Martín-Alonso et al., 2015) and a promotive effect on tumour cells (Paye et al., 2014). Additionally, it was shown that its expression was much higher in EOC patients than in pericarcinomatous tissues (Xiao et al., 2022). *PI3*, also known as elafin, is an elastase-specific inhibitor that directly affects tumour suppression by inhibiting elastase (Hunt et al., 2013). High levels of *PI3* are associated with severe disease severity in various cancers (Hunt et al., 2013; Longatto-Filho et al., 2021), while another TCGA-OV analysis showed its prognostic value in OC (Li J et al., 2020). Additionally, high elafin expression has been associated with unfavorable OS but better immunotherapy responses (Lu et al., 2023). Recent studies have found that *TGFBI* CpG islands are hypermethylated in adjacent normal colon tissue, with the corresponding sequences showing hypomethylation in colon cancer tissue, and that higher *TGFBI* levels are associated with poorer prognosis (Zhang H. et al., 2019). In mammals, Tolloid-like (mTLL)-1 is a BMP-1-associated protease, and *BMP1/TLL1* is involved in the process of tissue remodelling in the ovary, assisting in the maturation of pre-collagen molecules and the deposition of collagen fibres (Ohnishi et al., 2005). In hepatocellular carcinoma (Matsuura et al., 2017), S*NP* may impact the splicing of *TLL1* mRNA and result in short variants with high catalytic activity, speeding up the development of liver fibrosis and cancer. In a recent study, *TGFBI, PI3, TLL1* and *MMP17* were predicted to be among the 19 genes that comprise the TME-related high grade serous ovarian carcinoma prognostic genetic panel (Belotti et al., 2022). *ANGPTL4* is regulated by peroxisome proliferator-activated receptor γ (PPARγ) (Aryal et al., 2019), who has been observed to be significantly increased in malignant ovarian tumours (grade 1, 2 and 3) compared to benign and borderline tumours (Zhang et al., 2005). It was also recently identified in a scRNA-seq study of ovarian cancer CAF ligands to epithelial cells (Carvalho et al., 2022). Finally, *ANGPTL4* and *TGFBI* were identified both in a hypoxia risk model constructed to reflect the OC immune microenvironment in and predict prognosis (Wei et al., 2021), and among the genes that comprise an OC glycolysis-related gene signature (Zhang et al., 2021).

To further assess the relationship between the ECM risk score and the immune microenvironment, we assessed the abundance of multiple inflitrating cells in the immune microenvironment of these patients using multiple algorithms. Patients with more infiltrating and activated immune cells in TME may have better immunotherapeutic outcomes (Li et al., 2016). We observed higher levels of CD8 T cell infiltration in the low-risk group, suggesting enhanced immune surveillance via CD8^+^ T cells, while this implies a possible enhanced susceptibility to PD-1/PD-L1-targeted immune checkpoint therapies (Strickland et al., 2016; Iyer et al., 2021). Furthermore, the degree of macrophage infiltration was similar to both CD8 and CD4 T cell infiltration, with both showing high levels in the low-risk group. This is consistent with previous studies describing a positive correlation between T cells and macrophage infiltration levels (Desbois et al., 2020).

Previous studies have also shown that cancer cells regulate their local microenvironment to promote tumour survival, chemoresistance and evasion of immune surveillance (Kim et al., 2007) and that there is a tight association between malignant tumour cells and CAFs in promoting tumour growth and survival (Xing et al., 2010; Karagiannis et al., 2012; Chen et al., 2021). scRNA-seq analysis of high grade serous ovarian cancers, also showed that CAFs induce epithelial-mesenchymal transition (EMT) of tumor cells via TGFβ signaling, with consequent effects on chemoresistance and metastasis (Kan et al., 2020). In accordance with these, our results also showed that higher levels of CAFs were found in high-risk groups with poor prognosis.

It is known that the immune microenvironment immune cells, immunomodulating factors and immune checkpoint molecules are crucial for the immune escape of tumour cells (Charoentong et al., 2017; Zhang Y. et al., 2019). We thus developed an immunophenoscore (IPS) based on immune subpopulation infiltration and expression of immune regulatory molecules using the random forest to identify determinants of immunogenicity. Among several IPS subtypes tested, we found that the low-risk group had higher IPS and could benefit during treatment with immune checkpoint inhibitors. As the immunophenoscore is a surrogate to patients’ immunotherapeutic outcomes, our IPS results of the ECMs risk scores may only be considered of predictive value, and future studies will confirm their clinical importance.

We also explored the tumour mutation burden (TMB) changes in the TCGA-OV cohort. The ovarian cancer genome exhibits high levels of instability, as evidenced by functional cells (Stewart et al., 2011), copy number changes (Schwarz et al., 2015), and status of somatic mutations (Bashashati et al., 2013). TMB is the total number of substitutions and insertion/deletion mutations per megabase in the exon-coding region of the gene under evaluation in the tumour cell genome (Stenzinger et al., 2019). Somatic mutations may result in tumourigenesis and many somatic mutations can generate neoantigens facilitating anti-tumour immunity (Gubin et al., 2015). In a study on immunotherapy for lung cancer, researchers discovered that patients with PD-L1 1% but a subgroup of 10 mutations/Mb in the combination chemotherapy group had a better objective response rate (ORR) and median progression-free survival (median PFS, mPFS) with the immune combination regimen CheckMate 227 (Hellmann et al., 2018). This suggests that in the higher TMB population, PFS was better in the combination immunotherapy group than in the chemotherapy alone group, irrespective of PD-L1 expression. In our study, the mean TMB values were higher in the low-risk group than in the high-risk group, implying that the low-risk group may have more potential for immunotherapy. *TP53*, the gene encoding the tumour suppressor protein p53, is one of the most commonly mutated genes in human cancers, and driver mutations are prevalent in high-grade ovarian plasmacytoma (Ahmed et al., 2010). Chalmers and coworkers have shown that *TP53* mutations were associated with high TMB (Chalmers et al., 2017). Our study similarly confirmed that in the TCGA-OV datasets *TP53* mutations were the most frequent in the high- and low-risk groups, and that *TP53* mutations were higher in the low-risk group than in the high-risk group. However, there are limitations to cohort-based studies. Most mutated genes are unique to each case, and in clinical treatment, patients should be treated based on their mutation sequencing results. Our analysis may provide theoretical support for the selection of immunological agents.

Our study aimed to investigate the role of the matrisome and the gene changes in the ECM in a widely studied, publicly available ovarian cancer transcriptomic and clinicopathological collection of patients. The role of matrisome has been regretfully understudied in this type or cancer that commonly goes undetected till it reaches high grades, as the ECM can influence drug resistance. We used bioinformatics and machine learning methods to investigate the TCGA-OV collection and identified several prognostic genes, some of which have also been identified by previous studies. Given the current cost-effectiveness of biotechnological approaches, rapid genetic testing tools are commonly promoted and widely applied in clinical diagnostics and treatment (Young and Argáez, 2019). These tools have improved accuracy and testing times have significantly shortened. Targeted multigene tests and genetic screening can be thus rapidly employed to assist diagnosis postoperatively and develop more effective treatment plans.

## Conclusion

In conclusion, this study developed an ECM risk score prediction model to enable prognosis of patients with ovarian serous adenocarcinoma. We further identified the tumour microenvironment and somatic mutations using the TCGA-OV collection datasets. These results should be further validated with targeted future studies to evaluate their real predictive function and their use in personalized immunotherapy applications.

## Data Availability

Publicly available datasets were analyzed in this study. This data can be found here: https://xenabrowser.net/datapages/?cohort&equals;GDC%20TCGA%20Ovarian%20Cancer%20(OV)&removeHub&equals;https%3A%2F%2Fxena.treehouse.gi.ucsc.edu%3A443.

## Supplementary material

SUPPLEMENTARY FIGURE S1 Enrichment analysis for different expression genes **(A)** GOKEGG results of TCGA-OV. Top 5 Head **(B)** and Tail **(C)** aggregation of GSEA results in TCGA-OV DEGs.

SUPPLEMENTARY FIGURE S2 Comparison of immune cell infiltration between high/low ECM risk score group groups in TCGA-OV.

SUPPLEMENTARY FIGURE S3 Summary of somatic mutation analysis in high/low ECM risk score group in TCGAOV Summary of mutations in the high **(A)** and low **(B)** ECM risk score groups. Mutation profile of TCGAOV patients in high **(C)** and low **(D)** ECM risk score group and percentage of top 6 mutated genes in high/low ECM risk score group **(E)**. Comparison of mutation load among high/low ECM risk score group and TCGA cohorts **(F)**.

SUPPLEMENTARY FIGURE S4 Details of ECM risk score in the 15 key gene mutations.

SUPPLEMENTARY TABLE S1 ECM-related gene list.

SUPPLEMENTARY TABLE S2 Drug-gene interactions.
