# Supplementary figures and images for: An artificial intelligence prediction model based on extracellular matrix proteins for the prognostic prediction and immunotherapeutic evaluation of ovarian serous adenocarcinoma
Source: Front Mol Biosci. 2023 Jun 14;10:1200354. doi: 10.3389/fmolb.2023.1200354 (PMC10301747; doi:10.3389/fmolb.2023.1200354)

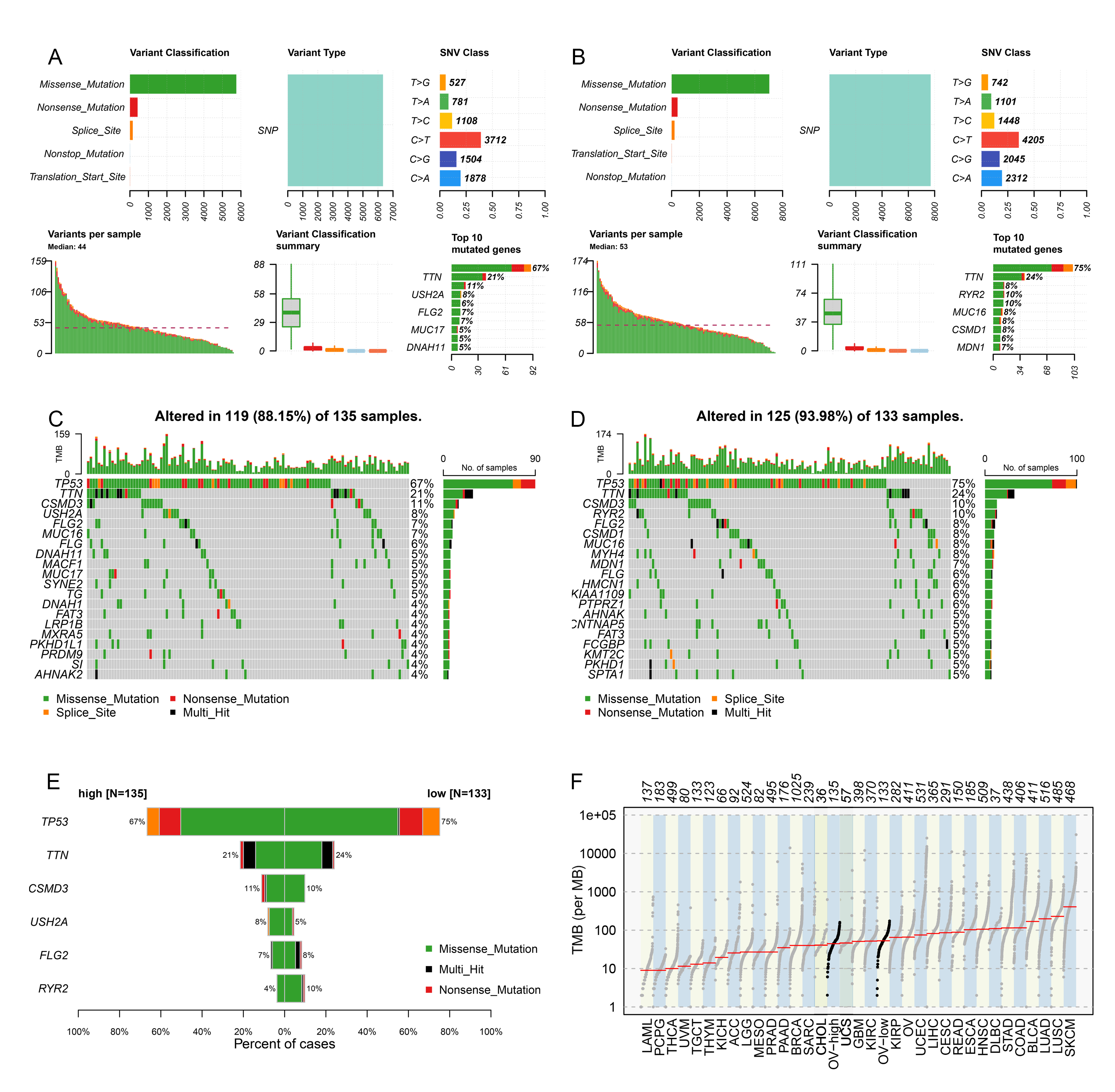

Supplement: Supplementary file 2 [file Image3.TIF]

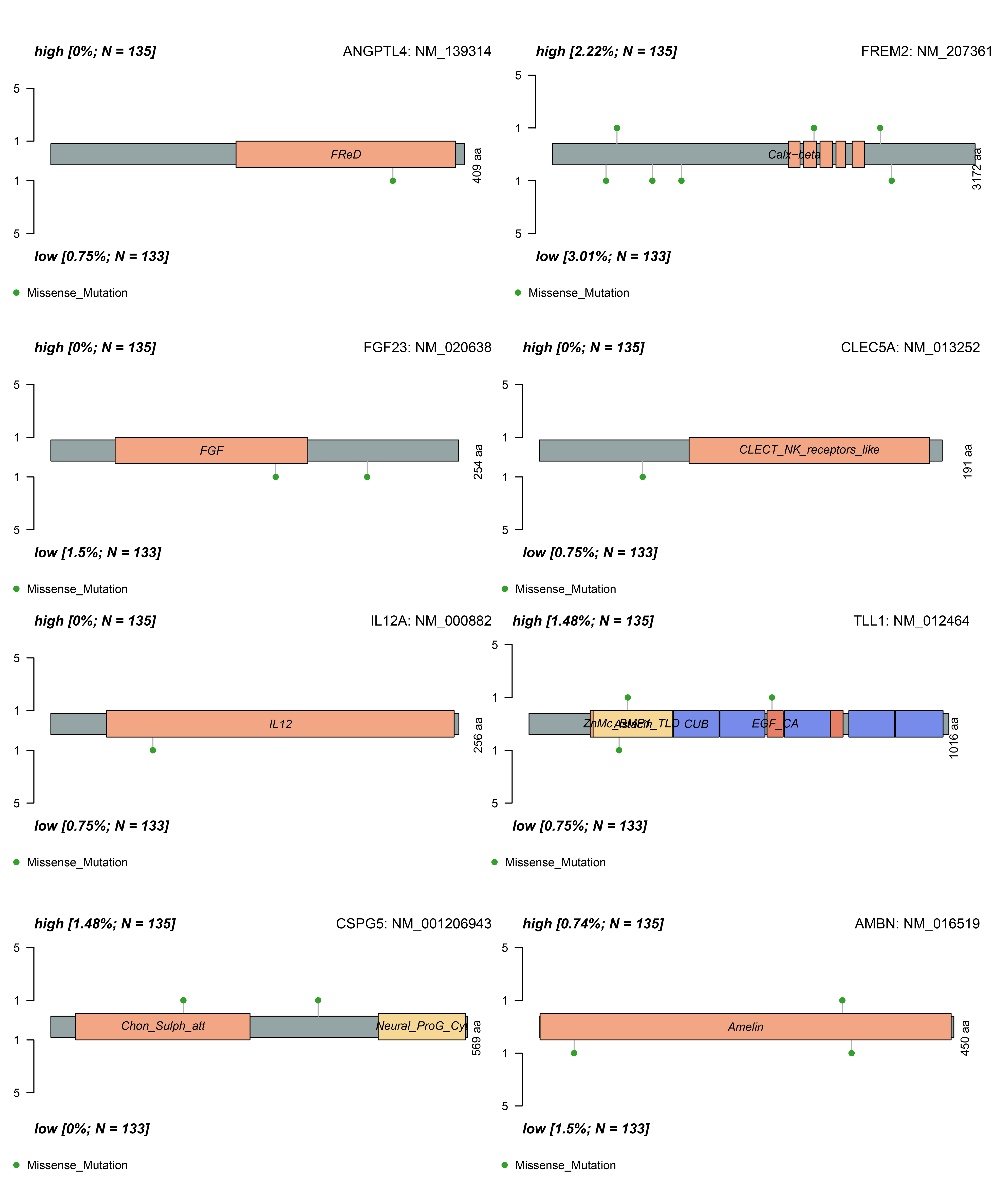

Supplement: Supplementary file 3 [file Image4.TIF]

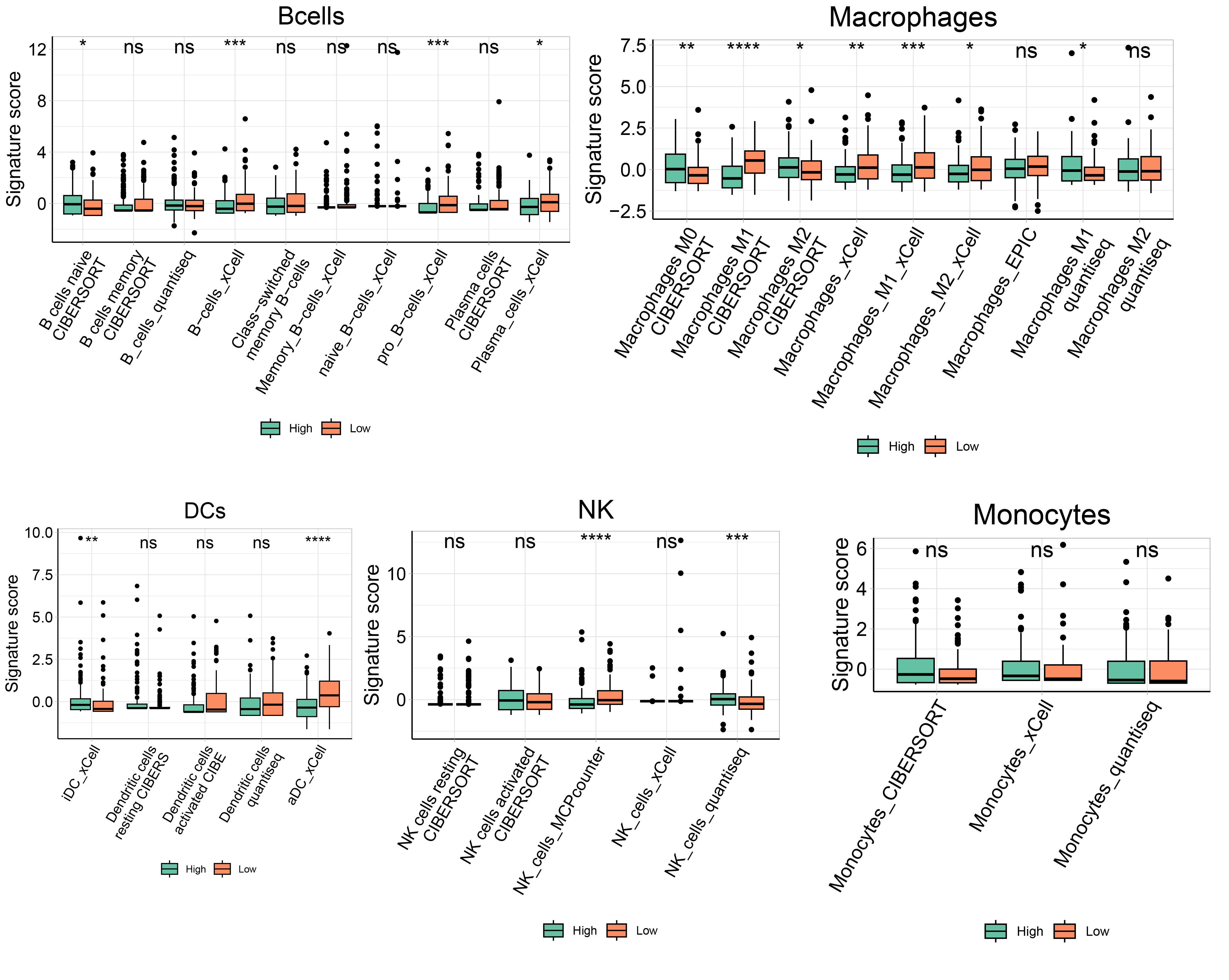

Supplement: Supplementary file 4 [file Image2.TIF]
