# Supplementary material for: An artificial intelligence prediction model based on extracellular matrix proteins for the prognostic prediction and immunotherapeutic evaluation of ovarian serous adenocarcinoma
Source: Front Mol Biosci. 2023 Jun 14;10:1200354. doi: 10.3389/fmolb.2023.1200354 (PMC10301747; doi:10.3389/fmolb.2023.1200354)
Supplement: Supplementary file 6 [file Image1.pdf]

# Figure S1 Enrichment analysis for different expression genes

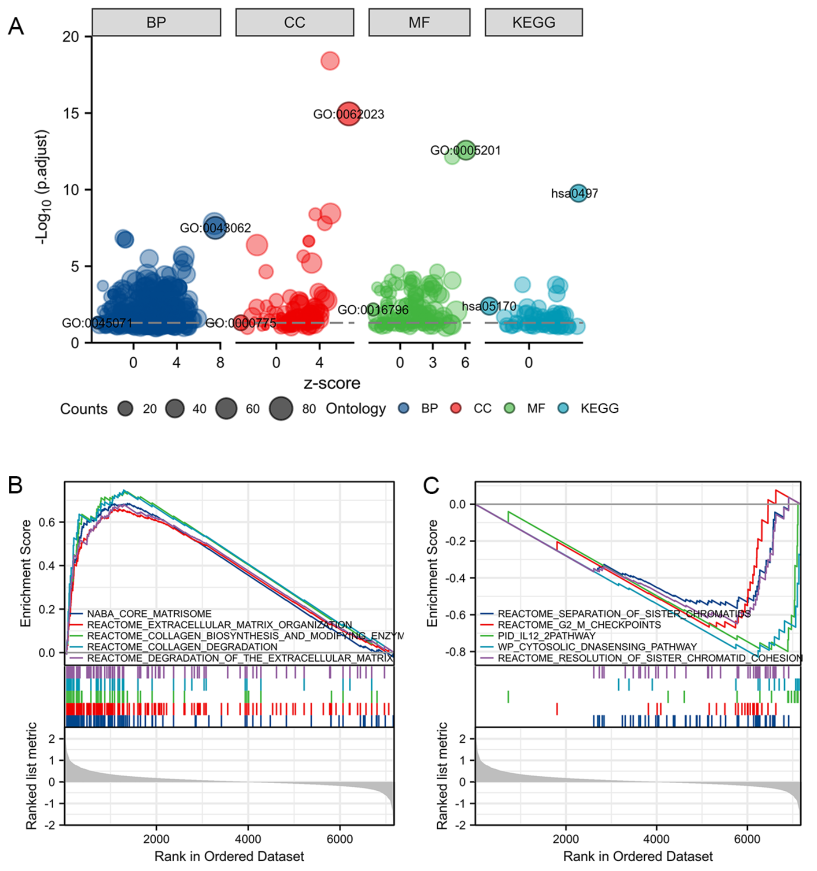

Figure S2 Comparison of immune cell infiltration between high/low ECM risk score group groups in TCGA-OV

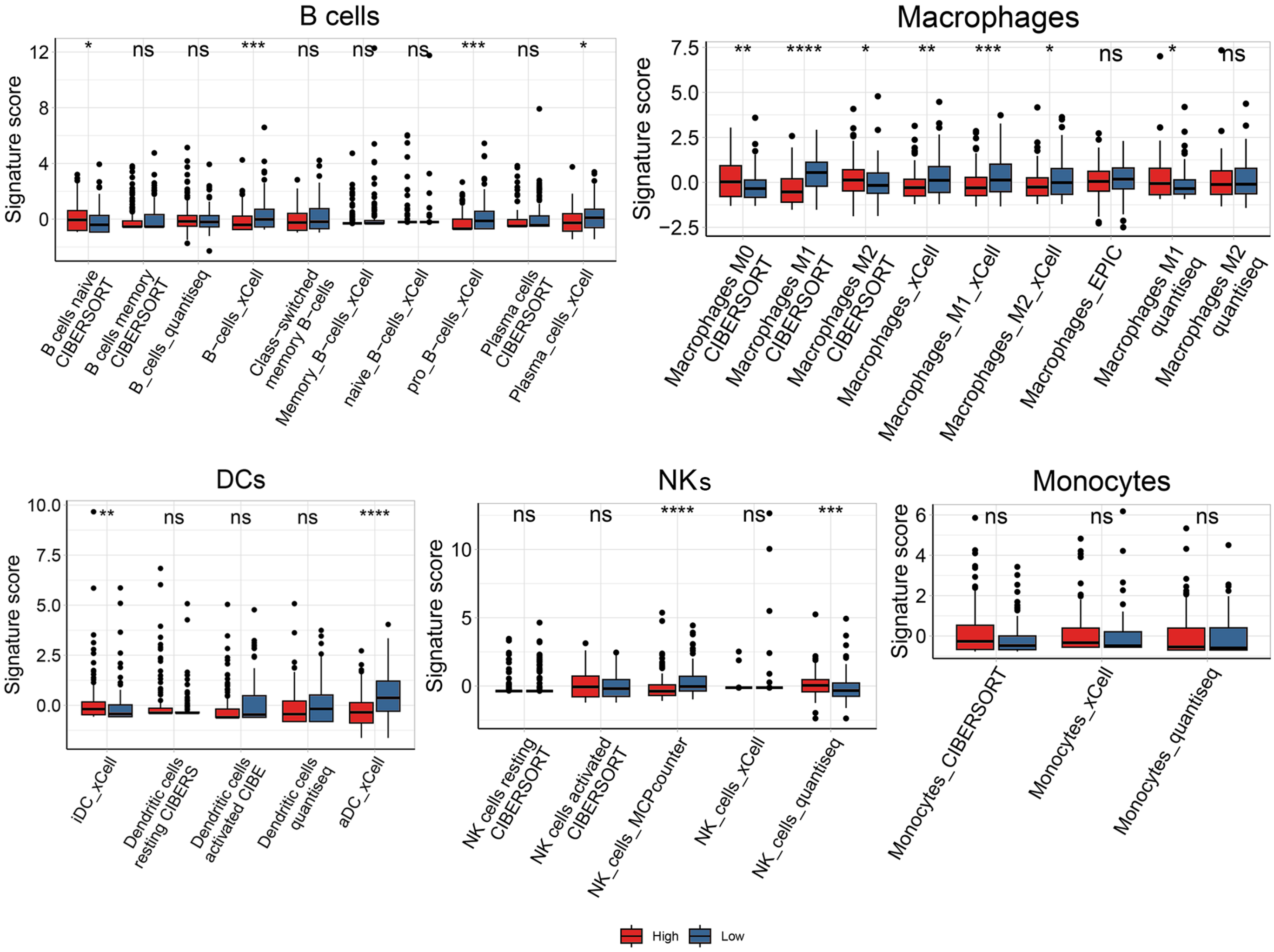

Figure S3 Summary of somatic mutation analysis in high/low ECM risk score group in TCGA-OV

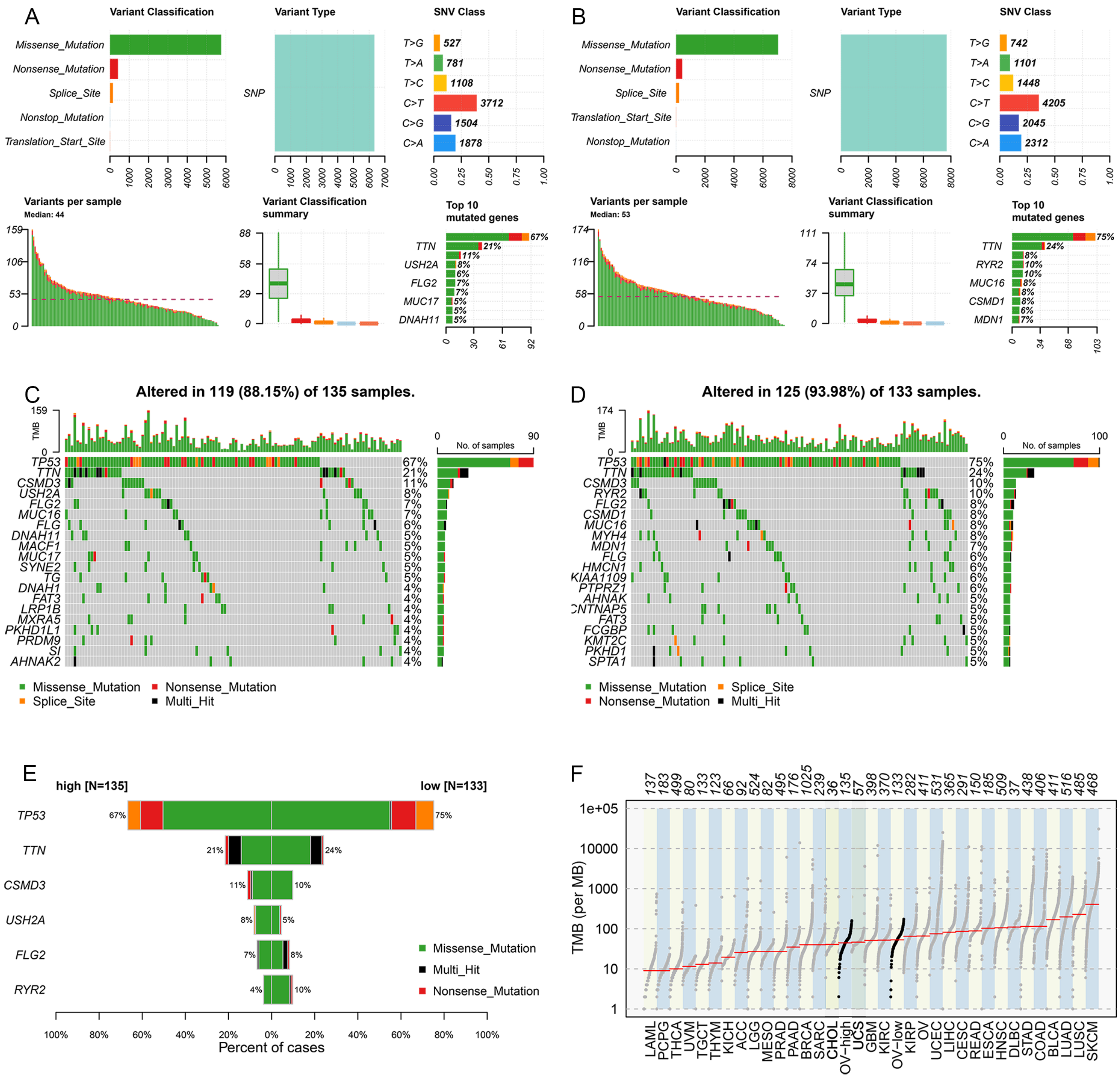

# Figure S4 Details of ECM risk score in the 15 key gene mutations

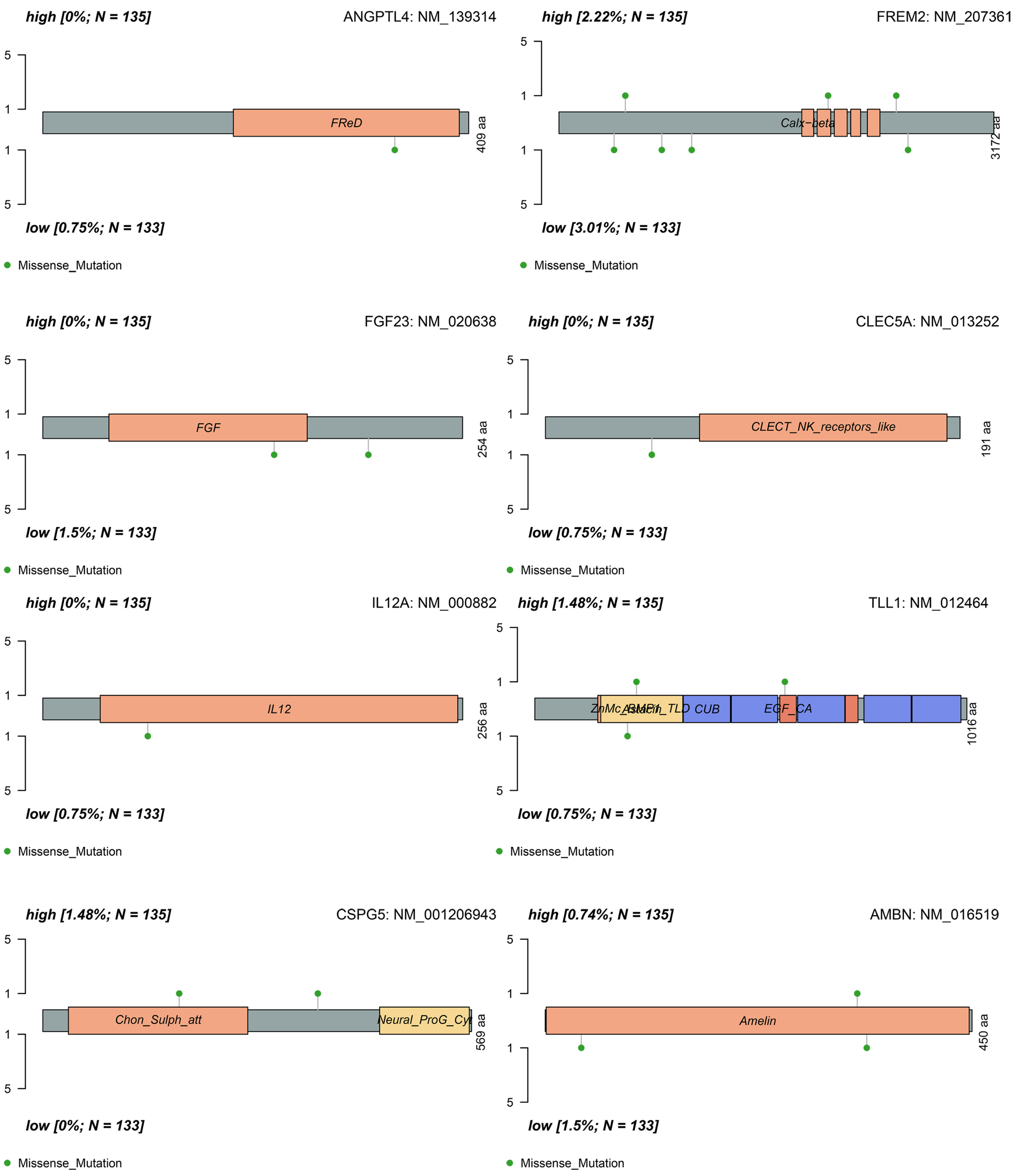

Table S1 ECM-related gene list.

| Gene Symbol | Gene Name |
|---|---|
| ABI3BP | ABI family, member 3 (NESH) binding protein |
| ADIPOQ | adiponectin, C1Q and collagen domain containing |
| AEBP1 | AE binding protein 1 |
| AGRN | agrin |
| AMBN | ameloblastin (enamel matrix protein) |
| AMELX | amelogenin (amelogenesis imperfecta 1, X-linked) |
| AMELY | amelogenin, Y-linked |
| BGLAP | bone gamma-carboxyglutamate (gla) protein |
| BMPER | BMP binding endothelial regulator |
| BSPH1 | binder of sperm protein homolog 1 |
| CDCP2 | CUB domain containing protein 2 |
| CILP | cartilage intermediate layer protein, nucleotide pyrophosphohydrolase |
| CILP2 | cartilage intermediate layer protein 2 |
| COCH | coagulation factor C homolog, cochlin (Limulus polyphemus) |
| COLQ | collagen-like tail subunit (single strand of homotrimer) of asymmetric acetylcholinesterase |
| COMP | cartilage oligomeric matrix protein |
| CRELD1 | cysteine-rich with EGF-like domains 1 |
| CRELD2 | cysteine-rich with EGF-like domains 2 |
| CRIM1 | cysteine rich transmembrane BMP regulator 1 (chordin-like) |
| CRISPLD1 | cysteine-rich secretory protein LCCL domain containing 1 |
| CRISPLD2 | cysteine-rich secretory protein LCCL domain containing 2 |
| CTGF | connective tissue growth factor |
| CTHRC1 | collagen triple helix repeat containing 1 |
| CYR61 | cysteine-rich, angiogenic inducer, 61 |
| DDX26B | DEAD/H (Asp-Glu-Ala-Asp/His) box polypeptide 26B |
| DMBT1 | deleted in malignant brain tumors 1 |
| DMP1 | dentin matrix acidic phosphoprotein 1 |
| DPT | dermatopontin |
| DSPP | dentin sialophosphoprotein |
| ECM1 | extracellular matrix protein 1 |
| ECM2 | extracellular matrix protein 2, female organ and adipocyte specific |
| EDIL3 | EGF-like repeats and discoidin I-like domains 3 |
| EFEMP1 | EGF-containing fibulin-like extracellular matrix protein 1 |
| EFEMP2 | EGF-containing fibulin-like extracellular matrix protein 2 |
| EGFLAM | EGF-like, fibronectin type III and laminin G domains |
| ELN | elastin |
| ELSPBP1 | epididymal sperm binding protein 1 |
| EMID1 | EMI domain containing 1 |
| EMILIN1 | elastin microfibril interfacer 1 |
| EMILIN2 | elastin microfibril interfacer 2 |
| EMILIN3 | elastin microfibril interfacer 3 |
| EYS | eyes shut homolog (Drosophila) |
| FBLN1 | fibulin 1 |
| FBLN2 | fibulin 2 |
| FBLN5 | fibulin 5 |
| FBLN7 | fibulin 7 |
| FBN1 | fibrillin 1 |
| FBN2 | fibrillin 2 |
| FBN3 | fibrillin 3 |
| FGA | fibrinogen alpha chain |

| | |
|---|---|
| FGB | fibrinogen beta chain |
| FGG | fibrinogen gamma chain |
| FGL1 | fibrinogen-like 1 |
| FGL2 | fibrinogen-like 2 |
| FN1 | fibronectin 1 |
| FNDC1 | fibronectin type III domain containing 1 |
| FNDC7 | fibronectin type III domain containing 7 |
| FNDC8 | fibronectin type III domain containing 8 |
| FRAS1 | Fraser syndrome 1 |
| GAS6 | growth arrest-specific 6 |
| GLDN | gliomedin |
| HMCN1 | hemicentin 1 |
| HMCN2 | hemicentin 2 |
| IBSP | integrin-binding sialoprotein |
| IGFALS | insulin-like growth factor binding protein, acid labile subunit |
| IGFBP1 | insulin-like growth factor binding protein 1 |
| IGFBP2 | insulin-like growth factor binding protein 2, 36kDa |
| IGFBP3 | insulin-like growth factor binding protein 3 |
| IGFBP4 | insulin-like growth factor binding protein 4 |
| IGFBP5 | insulin-like growth factor binding protein 5 |
| IGFBP6 | insulin-like growth factor binding protein 6 |
| IGFBP7 | insulin-like growth factor binding protein 7 |
| IGFBPL1 | insulin-like growth factor binding protein-like 1 |
| IGSF10 | immunoglobulin superfamily, member 10 |
| KAL1 | Kallmann syndrome 1 sequence |
| KCP | kielin/chordin-like protein |
| LAMA1 | laminin, alpha 1 |
| LAMA2 | laminin, alpha 2 |
| LAMA3 | laminin, alpha 3 |
| LAMA4 | laminin, alpha 4 |
| LAMA5 | laminin, alpha 5 |
| LAMB1 | laminin, beta 1 |
| LAMB2 | laminin, beta 2 (laminin S) |
| LAMB3 | laminin, beta 3 |
| LAMB4 | laminin, beta 4 |
| LAMC1 | laminin, gamma 1 (formerly LAMB2) |
| LAMC2 | laminin, gamma 2 |
| LAMC3 | laminin, gamma 3 |
| LGI1 | leucine-rich, glioma inactivated 1 |
| LGI2 | leucine-rich repeat LGI family, member 2 |
| LGI3 | leucine-rich repeat LGI family, member 3 |
| LGI4 | leucine-rich repeat LGI family, member 4 |
| LRG1 | leucine-rich alpha-2-glycoprotein 1 |
| LTBP1 | latent transforming growth factor beta binding protein 1 |
| LTBP2 | latent transforming growth factor beta binding protein 2 |
| LTBP3 | latent transforming growth factor beta binding protein 3 |
| LTBP4 | latent transforming growth factor beta binding protein 4 |
| MATN1 | matrilin 1, cartilage matrix protein |
| MATN2 | matrilin 2 |
| MATN3 | matrilin 3 |
| MATN4 | matrilin 4 |
| MEPE | matrix extracellular phosphoglycoprotein |

| | |
|---|---|
| MFAP1 | microfibrillar-associated protein 1 |
| MFAP2 | microfibrillar-associated protein 2 |
| MFAP3 | microfibrillar-associated protein 3 |
| MFAP4 | microfibrillar-associated protein 4 |
| MFAP5 | microfibrillar associated protein 5 |
| MFGE8 | milk fat globule-EGF factor 8 protein |
| MGP | matrix Gla protein |
| MMRN1 | multimerin 1 |
| MMRN2 | multimerin 2 |
| MXRA5 | matrix-remodelling associated 5 |
| NDNF | chromosome 4 open reading frame 31 |
| NELL1 | NEL-like 1 (chicken) |
| NELL2 | NEL-like 2 (chicken) |
| NID1 | nidogen 1 |
| NID2 | nidogen 2 (osteonidogen) |
| NOV | nephroblastoma overexpressed gene |
| NPNT | nephronectin |
| NTN1 | netrin 1 |
| NTN3 | netrin 3 |
| NTN4 | netrin 4 |
| NTN5 | netrin 5 |
| NTNG1 | netrin G1 |
| NTNG2 | netrin G2 |
| OIT3 | oncoprotein induced transcript 3 |
| OTOG | otogelin |
| OTOL1 | otolin-1 |
| PAPLN | papilin, proteoglycan-like sulfated glycoprotein |
| PCOLCE | procollagen C-endopeptidase enhancer |
| PCOLCE2 | procollagen C-endopeptidase enhancer 2 |
| POMZP3 | POM (POM121 homolog, rat) and ZP3 fusion |
| POSTN | periostin, osteoblast specific factor |
| PXDN | peroxidasin homolog (Drosophila) |
| PXDNL | peroxidasin homolog (Drosophila)-like |
| RELN | reelin |
| RSPO1 | R-spondin homolog (Xenopus laevis) |
| RSPO2 | R-spondin 2 homolog (Xenopus laevis) |
| RSPO3 | R-spondin 3 homolog (Xenopus laevis) |
| RSPO4 | R-spondin family, member 4 |
| SBSPON | chromosome 8 open reading frame 84 |
| SLIT1 | slit homolog 1 (Drosophila) |
| SLIT2 | slit homolog 2 (Drosophila) |
| SLIT3 | slit homolog 3 (Drosophila) |
| SMOC1 | SPARC related modular calcium binding 1 |
| SMOC2 | SPARC related modular calcium binding 2 |
| SNED1 | sushi, nidogen and EGF-like domains 1 |
| SPARC | secreted protein, acidic, cysteine-rich (osteonectin) |
| SPARCL1 | SPARC-like 1 (hevin) |
| SPON1 | spondin 1, extracellular matrix protein |
| SPON2 | spondin 2, extracellular matrix protein |
| SPP1 | secreted phosphoprotein 1 |
| SRPX | sushi-repeat-containing protein, X-linked |
| SRPX2 | sushi-repeat-containing protein, X-linked 2 |

| | |
|---|---|
| SSPO | SCO-spondin homolog (Bos taurus) |
| SVEP1 | sushi, von Willebrand factor type A, EGF and pentraxin domain containing 1 |
| TECTA | tectorin alpha |
| TECTB | tectorin beta |
| TGFBI | transforming growth factor, beta-induced, 68kDa |
| THBS1 | thrombospondin 1 |
| THBS2 | thrombospondin 2 |
| THBS3 | thrombospondin 3 |
| THBS4 | thrombospondin 4 |
| THSD4 | thrombospondin, type I, domain containing 4 |
| TINAG | tubulointerstitial nephritis antigen |
| TINAGL1 | tubulointerstitial nephritis antigen-like 1 |
| TNC | tenascin C |
| TNFAIP6 | tumor necrosis factor, alpha-induced protein 6 |
| TNN | tenascin N |
| TNR | tenascin R (restrictin, janusin) |
| TNXB | tenascin XB |
| TSKU | tsukushi small leucine rich proteoglycan homolog (Xenopus laevis) |
| TSPEAR | chromosome 21 open reading frame 29 |
| VIT | vitrin |
| VTN | vitronectin |
| VWA1 | von Willebrand factor A domain containing 1 |
| VWA2 | von Willebrand factor A domain containing 2 |
| VWA3A | von Willebrand factor A domain containing 3A |
| VWA3B | von Willebrand factor A domain containing 3B |
| VWA5A | von Willebrand factor A domain containing 5A |
| VWA5B1 | von Willebrand factor A domain containing 5B1 |
| VWA5B2 | von Willebrand factor A domain containing 5B2 |
| VWA7 | chromosome 6 open reading frame 27 |
| VWA9 | chromosome 15 open reading frame 44 |
| VWCE | von Willebrand factor C and EGF domains |
| VWDE | von Willebrand factor D and EGF domains |
| VWF | von Willebrand factor |
| WISP1 | WNT1 inducible signaling pathway protein 1 |
| WISP2 | WNT1 inducible signaling pathway protein 2 |
| WISP3 | WNT1 inducible signaling pathway protein 3 |
| ZP1 | zona pellucida glycoprotein 1 (sperm receptor) |
| ZP2 | zona pellucida glycoprotein 2 (sperm receptor) |
| ZP3 | zona pellucida glycoprotein 3 (sperm receptor) |
| ZP4 | zona pellucida glycoprotein 4 |
| ZPLD1 | zona pellucida-like domain containing 1 |
| COL10A1 | collagen, type X, alpha 1 |
| COL11A1 | collagen, type XI, alpha 1 |
| COL11A2 | collagen, type XI, alpha 2 |
| COL12A1 | collagen, type XII, alpha 1 |
| COL13A1 | collagen, type XIII, alpha 1 |
| COL14A1 | collagen, type XIV, alpha 1 |
| COL15A1 | collagen, type XV, alpha 1 |
| COL16A1 | collagen, type XVI, alpha 1 |
| COL17A1 | collagen, type XVII, alpha 1 |
| COL18A1 | collagen, type XVIII, alpha 1 |

| | |
|---|---|
| COL19A1 | collagen, type XIX, alpha 1 |
| COL1A1 | collagen, type I, alpha 1 |
| COL1A2 | collagen, type I, alpha 2 |
| COL20A1 | collagen, type XX, alpha 1 |
| COL21A1 | collagen, type XXI, alpha 1 |
| COL22A1 | collagen, type XXII, alpha 1 |
| COL23A1 | collagen, type XXIII, alpha 1 |
| COL24A1 | collagen, type XXIV, alpha 1 |
| COL25A1 | collagen, type XXV, alpha 1 |
| COL26A1 | EMI domain containing 2 |
| COL27A1 | collagen, type XXVII, alpha 1 |
| COL28A1 | collagen, type XXVIII, alpha 1 |
| COL2A1 | collagen, type II, alpha 1 |
| COL3A1 | collagen, type III, alpha 1 |
| COL4A1 | collagen, type IV, alpha 1 |
| COL4A2 | collagen, type IV, alpha 2 |
| COL4A3 | collagen, type IV, alpha 3 (Goodpasture antigen) |
| COL4A4 | collagen, type IV, alpha 4 |
| COL4A5 | collagen, type IV, alpha 5 |
| COL4A6 | collagen, type IV, alpha 6 |
| COL5A1 | collagen, type V, alpha 1 |
| COL5A2 | collagen, type V, alpha 2 |
| COL5A3 | collagen, type V, alpha 3 |
| COL6A1 | collagen, type VI, alpha 1 |
| COL6A2 | collagen, type VI, alpha 2 |
| COL6A3 | collagen, type VI, alpha 3 |
| COL6A5 | collagen, type XXIX, alpha 1 |
| COL6A6 | collagen, type VI, alpha 6 |
| COL7A1 | collagen, type VII, alpha 1 |
| COL8A1 | collagen, type VIII, alpha 1 |
| COL8A2 | collagen, type VIII, alpha 2 |
| COL9A1 | collagen, type IX, alpha 1 |
| COL9A2 | collagen, type IX, alpha 2 |
| COL9A3 | collagen, type IX, alpha 3 |
| ACAN | aggrecan |
| ASPN | asporin |
| BCAN | brevican |
| BGN | biglycan |
| CHAD | chondroadherin |
| CHADL | chondroadherin-like |
| DCN | decorin |
| EPYC | epiphycan |
| ESM1 | endothelial cell-specific molecule 1 |
| FMOD | fibromodulin |
| HAPLN1 | hyaluronan and proteoglycan link protein 1 |
| HAPLN2 | hyaluronan and proteoglycan link protein 2 |
| HAPLN3 | hyaluronan and proteoglycan link protein 3 |
| HAPLN4 | hyaluronan and proteoglycan link protein 4 |
| HSPG2 | heparan sulfate proteoglycan 2 |
| IMPG1 | interphotoreceptor matrix proteoglycan 1 |
| IMPG2 | interphotoreceptor matrix proteoglycan 2 |
| KERA | keratocan |

| | |
|---|---|
| LUM | lumican |
| NCAN | neurocan |
| NYX | nyctalopin |
| OGN | osteoglycin |
| OMD | osteomodulin |
| OPTC | opticin |
| PODN | podocan |
| PODNL1 | podocan-like 1 |
| PRELP | proline/arginine-rich end leucine-rich repeat protein |
| PRG2 | proteoglycan 2, bone marrow (natural killer cell activator, eosinophil granule major basic protein) |
| PRG3 | proteoglycan 3 |
| PRG4 | p53-responsive gene 4 |
| SPOCK1 | sparc/osteonectin, cwcv and kazal-like domains proteoglycan (testican) 1 |
| SPOCK2 | sparc/osteonectin, cwcv and kazal-like domains proteoglycan (testican) 2 |
| SPOCK3 | sparc/osteonectin, cwcv and kazal-like domains proteoglycan (testican) 3 |
| SRGN | serglycin |
| VCAN | versican |
| ANXA1 | annexin A1 |
| ANXA10 | annexin A10 |
| ANXA11 | annexin A11 |
| ANXA13 | annexin A13 |
| ANXA2 | annexin A2 |
| ANXA3 | annexin A3 |
| ANXA4 | annexin A4 |
| ANXA5 | annexin A5 |
| ANXA6 | annexin A6 |
| ANXA7 | annexin A7 |
| ANXA8 | annexin A8 |
| ANXA8L1 | annexin A8-like 1 |
| ANXA9 | annexin A9 |
| C1QA | complement component 1, q subcomponent, A chain |
| C1QB | complement component 1, q subcomponent, B chain |
| C1QC | complement component 1, q subcomponent, C chain |
| C1QL1 | complement component 1, q subcomponent-like 1 |
| C1QL2 | complement component 1, q subcomponent-like 2 |
| C1QL3 | complement component 1, q subcomponent-like 3 |
| C1QL4 | complement component 1, q subcomponent-like 4 |
| C1QTNF1 | C1q and tumor necrosis factor related protein 1 |
| C1QTNF2 | C1q and tumor necrosis factor related protein 2 |
| C1QTNF3 | C1q and tumor necrosis factor related protein 3 |
| C1QTNF4 | C1q and tumor necrosis factor related protein 4 |
| C1QTNF5 | C1q and tumor necrosis factor related protein 5 |
| C1QTNF6 | C1q and tumor necrosis factor related protein 6 |
| C1QTNF7 | C1q and tumor necrosis factor related protein 7 |
| C1QTNF8 | C1q and tumor necrosis factor related protein 8 |
| C1QTNF9 | C1q and tumor necrosis factor related protein 9 |
| CD209 | CD209 molecule |
| CLC | Charcot-Leyden crystal protein |

| | |
|---|---|
| CLEC10A | C-type lectin domain family 10, member A |
| CLEC11A | C-type lectin domain family 11, member A |
| CLEC12A | C-type lectin domain family 12, member A |
| CLEC12B | C-type lectin domain family 12, member B |
| CLEC14A | C-type lectin domain family 14, member A |
| CLEC17A | C-type lectin domain family 17, member A |
| CLEC18A | C-type lectin domain family 18, member A |
| CLEC18B | C-type lectin domain family 18, member B |
| CLEC18C | C-type lectin domain family 18, member C |
| CLEC19A | C-type lectin domain family 19, member A |
| CLEC1A | C-type lectin domain family 1, member A |
| CLEC1B | C-type lectin domain family 1, member B |
| CLEC2A | C-type lectin domain family 2, member A |
| CLEC2B | C-type lectin domain family 2, member B |
| CLEC2D | C-type lectin domain family 2, member D |
| CLEC2L | C-type lectin domain family 2, member L |
| CLEC3A | C-type lectin domain family 3, member A |
| CLEC3B | C-type lectin domain family 3, member B |
| CLEC4A | C-type lectin domain family 4, member A |
| CLEC4C | C-type lectin domain family 4, member C |
| CLEC4D | C-type lectin domain family 4, member D |
| CLEC4E | C-type lectin domain family 4, member E |
| CLEC4F | C-type lectin domain family 4, member F |
| CLEC4G | C-type lectin domain family 4, member G |
| CLEC4M | C-type lectin domain family 4, member M |
| CLEC5A | C-type lectin domain family 5, member A |
| CLEC6A | C-type lectin domain family 6, member A |
| CLEC7A | C-type lectin domain family 7, member A |
| CLEC9A | C-type lectin domain family 9, member A |
| COLEC10 | collectin sub-family member 10 (C-type lectin) |
| COLEC11 | collectin sub-family member 11 |
| COLEC12 | collectin sub-family member 12 |
| CSPG4 | chondroitin sulfate proteoglycan 4 |
| CSPG5 | chondroitin sulfate proteoglycan 5 (neuroglycan C) |
| ELFN1 | extracellular leucine-rich repeat and fibronectin type III domain containing 1 |
| ELFN2 | extracellular leucine-rich repeat and fibronectin type III domain containing 2 |
| EMCN | endomucin |
| FCN1 | ficolin (collagen/fibrinogen domain containing) 1 |
| FCN2 | ficolin (collagen/fibrinogen domain containing lectin) 2 (hucolin) |
| FCN3 | ficolin (collagen/fibrinogen domain containing) 3 (Hakata antigen) |
| FREM1 | FRAS1 related extracellular matrix 1 |
| FREM2 | FRAS1 related extracellular matrix protein 2 |
| FREM3 | FRAS1 related extracellular matrix 3 |
| GPC1 | glypican 1 |
| GPC2 | glypican 2 |
| GPC3 | glypican 3 |
| GPC4 | glypican 4 |
| GPC5 | glypican 5 |
| GPC6 | glypican 6 |
| GREM1 | gremlin 1, cysteine knot superfamily, homolog (Xenopus laevis) |

| | |
|---|---|
| GRIFIN | galectin-related inter-fiber protein |
| HPX | hemopexin |
| HSPC159 | galectin-related protein |
| ITLN1 | intelectin 1 (galactofuranose binding) |
| ITLN2 | intelectin 2 |
| LGALS1 | lectin, galactoside-binding, soluble, 1 |
| LGALS12 | lectin, galactoside-binding, soluble, 12 |
| LGALS13 | lectin, galactoside-binding, soluble, 13 |
| LGALS14 | lectin, galactoside-binding, soluble, 14 |
| LGALS16 | beta-galactoside-binding lectin |
| LGALS2 | lectin, galactoside-binding, soluble, 2 |
| LGALS3 | lectin, galactoside-binding, soluble, 3 |
| LGALS4 | lectin, galactoside-binding, soluble, 4 |
| LGALS7 | lectin, galactoside-binding, soluble, 7 |
| LGALS8 | lectin, galactoside-binding, soluble, 8 |
| LGALS9 | lectin, galactoside-binding, soluble, 9 |
| LGALS9B | lectin, galactoside-binding, soluble, 9B |
| LGALS9C | lectin, galactoside-binding, soluble, 9C |
| LMAN1 | lectin, mannose-binding, 1 |
| LMAN1L | lectin, mannose-binding, 1 like |
| MBL2 | mannose-binding lectin (protein C) 2, soluble (opsonic defect) |
| MUC1 | mucin 1, cell surface associated |
| MUC12 | mucin 12, cell surface associated |
| MUC13 | mucin 13, cell surface associated |
| MUC15 | mucin 15, cell surface associated |
| MUC16 | mucin 16, cell surface associated |
| MUC17 | mucin 17, cell surface associated |
| MUC19 | mucin 19, oligomeric |
| MUC2 | mucin 2, oligomeric mucus/gel-forming |
| MUC20 | mucin 20, cell surface associated |
| MUC21 | mucin 21, cell surface associated |
| MUC22 | hypothetical LOC729792 |
| MUC3A | mucin 3A, cell surface associated |
| MUC4 | mucin 4, cell surface associated |
| MUC5AC | mucin 5AC, oligomeric mucus/gel-forming |
| MUC5B | mucin 5B, oligomeric mucus/gel-forming |
| MUC6 | mucin 6, oligomeric mucus/gel-forming |
| MUC7 | mucin 7, secreted |
| MUC8 | mucin 8 |
| MUCL1 | mucin-like 1 |
| OVGP1 | oviductal glycoprotein 1, 120kDa |
| PARM1 | prostate androgen-regulated mucin-like protein 1 |
| PLXDC1 | plexin domain containing 1 |
| PLXDC2 | plexin domain containing 2 |
| PLXNA1 | plexin A1 |
| PLXNA2 | plexin A2 |
| PLXNA3 | plexin A3 |
| PLXNA4 | plexin A4 |
| PLXNB1 | plexin B1 |
| PLXNB2 | plexin B2 |
| PLXNB3 | plexin B3 |
| PLXNC1 | plexin C1 |

| | |
|---|---|
| PLXND1 | plexin D1 |
| PROL1 | proline rich, lacrimal 1 |
| REG1A | regenerating islet-derived 1 alpha |
| REG1B | regenerating islet-derived 1 beta |
| REG3A | regenerating islet-derived 3 alpha |
| REG3G | regenerating islet-derived 3 gamma |
| REG4 | regenerating islet-derived family, member 4 |
| SDC1 | syndecan 1 |
| SDC2 | syndecan 2 |
| SDC3 | syndecan 3 |
| SDC4 | syndecan 4 |
| SEMA3A | sema domain, immunoglobulin domain (Ig), short basic domain, secreted, (semaphorin) 3A |
| SEMA3B | sema domain, immunoglobulin domain (Ig), short basic domain, secreted, (semaphorin) 3B |
| SEMA3C | sema domain, immunoglobulin domain (Ig), short basic domain, secreted, (semaphorin) 3C |
| SEMA3D | sema domain, immunoglobulin domain (Ig), short basic domain, secreted, (semaphorin) 3D |
| SEMA3E | sema domain, immunoglobulin domain (Ig), short basic domain, secreted, (semaphorin) 3E |
| SEMA3F | sema domain, immunoglobulin domain (Ig), short basic domain, secreted, (semaphorin) 3F |
| SEMA3G | sema domain, immunoglobulin domain (Ig), short basic domain, secreted, (semaphorin) 3G |
| SEMA4A | sema domain, immunoglobulin domain (Ig), transmembrane domain (TM) and short cytoplasmic domain, (semaphorin) 4A |
| SEMA4B | sema domain, immunoglobulin domain (Ig), transmembrane domain (TM) and short cytoplasmic domain, (semaphorin) 4B |
| SEMA4C | sema domain, immunoglobulin domain (Ig), transmembrane domain (TM) and short cytoplasmic domain, (semaphorin) 4C |
| SEMA4D | sema domain, immunoglobulin domain (Ig), transmembrane domain (TM) and short cytoplasmic domain, (semaphorin) 4D |
| SEMA4F | sema domain, immunoglobulin domain (Ig), transmembrane domain (TM) and short cytoplasmic domain, (semaphorin) 4F |
| SEMA4G | sema domain, immunoglobulin domain (Ig), transmembrane domain (TM) and short cytoplasmic domain, (semaphorin) 4G |
| SEMA5A | sema domain, seven thrombospondin repeats (type 1 and type 1-like), transmembrane domain (TM) and short cytoplasmic domain, (semaphorin) 5A |
| SEMA5B | sema domain, seven thrombospondin repeats (type 1 and type 1-like), transmembrane domain (TM) and short cytoplasmic domain, (semaphorin) 5B |
| SEMA6A | sema domain, transmembrane domain (TM), and cytoplasmic domain, (semaphorin) 6A |
| SEMA6B | sema domain, transmembrane domain (TM), and cytoplasmic domain, (semaphorin) 6B |
| SEMA6C | sema domain, transmembrane domain (TM), and cytoplasmic domain, (semaphorin) 6C |
| SEMA6D | sema domain, transmembrane domain (TM), and cytoplasmic domain, (semaphorin) 6D |

| | |
|---|---|
| SEMA7A | semaphorin 7A, GPI membrane anchor (John Milton Hagen blood group) |
| SFTA2 | surfactant associated 2 |
| SFTA3 | surfactant associated 3 |
| SFTPA1 | surfactant protein A1 |
| SFTPA2 | surfactant protein A2 |
| SFTPB | surfactant protein B |
| SFTPC | surfactant protein C |
| SFTPD | surfactant protein D |
| A2M | alpha-2-macroglobulin |
| A2ML1 | alpha-2-macroglobulin-like 1 |
| ADAM10 | ADAM metallopeptidase domain 10 |
| ADAM11 | ADAM metallopeptidase domain 11 |
| ADAM12 | ADAM metallopeptidase domain 12 |
| ADAM15 | ADAM metallopeptidase domain 15 |
| ADAM17 | ADAM metallopeptidase domain 17 |
| ADAM18 | ADAM metallopeptidase domain 18 |
| ADAM19 | ADAM metallopeptidase domain 19 (meltrin beta) |
| ADAM2 | ADAM metallopeptidase domain 2 |
| ADAM20 | ADAM metallopeptidase domain 20 |
| ADAM21 | ADAM metallopeptidase domain 21 |
| ADAM22 | ADAM metallopeptidase domain 22 |
| ADAM23 | ADAM metallopeptidase domain 23 |
| ADAM28 | ADAM metallopeptidase domain 28 |
| ADAM29 | ADAM metallopeptidase domain 29 |
| ADAM30 | ADAM metallopeptidase domain 30 |
| ADAM32 | ADAM metallopeptidase domain 32 |
| ADAM33 | ADAM metallopeptidase domain 33 |
| ADAM7 | ADAM metallopeptidase domain 7 |
| ADAM8 | ADAM metallopeptidase domain 8 |
| ADAM9 | ADAM metallopeptidase domain 9 (meltrin gamma) |
| ADAMDEC1 | ADAM-like, decysin 1 |
| ADAMTS1 | ADAM metallopeptidase with thrombospondin type 1 motif, 1 |
| ADAMTS10 | ADAM metallopeptidase with thrombospondin type 1 motif, 10 |
| ADAMTS12 | ADAM metallopeptidase with thrombospondin type 1 motif, 12 |
| ADAMTS13 | ADAM metallopeptidase with thrombospondin type 1 motif, 13 |
| ADAMTS14 | ADAM metallopeptidase with thrombospondin type 1 motif, 14 |
| ADAMTS15 | ADAM metallopeptidase with thrombospondin type 1 motif, 15 |
| ADAMTS16 | ADAM metallopeptidase with thrombospondin type 1 motif, 16 |
| ADAMTS17 | ADAM metallopeptidase with thrombospondin type 1 motif, 17 |
| ADAMTS18 | ADAM metallopeptidase with thrombospondin type 1 motif, 18 |
| ADAMTS19 | ADAM metallopeptidase with thrombospondin type 1 motif, 19 |
| ADAMTS2 | ADAM metallopeptidase with thrombospondin type 1 motif, 2 |
| ADAMTS20 | ADAM metallopeptidase with thrombospondin type 1 motif, 20 |
| ADAMTS3 | ADAM metallopeptidase with thrombospondin type 1 motif, 3 |
| ADAMTS4 | ADAM metallopeptidase with thrombospondin type 1 motif, 4 |
| ADAMTS5 | ADAM metallopeptidase with thrombospondin type 1 motif, 5 |
| ADAMTS6 | ADAM metallopeptidase with thrombospondin type 1 motif, 6 |
| ADAMTS7 | ADAM metallopeptidase with thrombospondin type 1 motif, 7 |
| ADAMTS8 | ADAM metallopeptidase with thrombospondin type 1 motif, 8 |
| ADAMTS9 | ADAM metallopeptidase with thrombospondin type 1 motif, 9 |
| ADAMTSL1 | ADAMTS-like 1 |

| | |
|---|---|
| ADAMTSL2 | ADAMTS-like 2 |
| ADAMTSL3 | ADAMTS-like 3 |
| ADAMTSL4 | ADAMTS-like 4 |
| ADAMTSL5 | ADAMTS-like 5 |
| AGT | angiotensinogen (serpin peptidase inhibitor, clade A, member 8) |
| AMBP | alpha-1-microglobulin/bikunin precursor |
| ASTL | astacin-like metallo-endopeptidase (M12 family) |
| BMP1 | bone morphogenetic protein 1 |
| C17orf58 | chromosome 17 open reading frame 58 |
| CD109 | CD109 molecule |
| CELA1 | chymotrypsin-like elastase family, member 1 |
| CELA2A | chymotrypsin-like elastase family, member 2A |
| CELA2B | chymotrypsin-like elastase family, member 2B |
| CELA3A | chymotrypsin-like elastase family, member 3A |
| CELA3B | chymotrypsin-like elastase family, member 3B |
| CPAMD8 | C3 and PZP-like, alpha-2-macroglobulin domain containing 8 |
| CPN2 | carboxypeptidase N, polypeptide 2 |
| CST1 | cystatin SN |
| CST11 | cystatin 11 |
| CST2 | cystatin SA |
| CST3 | cystatin C |
| CST4 | cystatin S |
| CST5 | cystatin D |
| CST6 | cystatin E/M |
| CST7 | cystatin F (leukocystatin) |
| CST8 | cystatin 8 (cystatin-related epididymal specific) |
| CST9 | cystatin 9 (testatin) |
| CST9L | cystatin 9-like |
| CSTA | cystatin A (stefin A) |
| CSTB | cystatin B (stefin B) |
| CSTL1 | cystatin-like 1 |
| CTSA | cathepsin A |
| CTSB | cathepsin B |
| CTSC | cathepsin C |
| CTSD | cathepsin D |
| CTSE | cathepsin E |
| CTSF | cathepsin F |
| CTSG | cathepsin G |
| CTSH | cathepsin H |
| CTSK | cathepsin K |
| CTSL | cathepsin L1 |
| CTSO | cathepsin O |
| CTSS | cathepsin S |
| CTSV | cathepsin L2 |
| CTSW | cathepsin W |
| CTSZ | cathepsin Z |
| EGLN1 | egl nine homolog 1 (C. elegans) |
| EGLN2 | egl nine homolog 2 (C. elegans) |
| EGLN3 | egl nine homolog 3 (C. elegans) |
| ELANE | elastase, neutrophil expressed |
| F10 | coagulation factor X |
| F12 | coagulation factor XII (Hageman factor) |

| | |
|---|---|
| F13A1 | coagulation factor XIII, A1 polypeptide |
| F13B | coagulation factor XIII, B polypeptide |
| F2 | coagulation factor II (thrombin) |
| F7 | coagulation factor VII (serum prothrombin conversion accelerator) |
| F9 | coagulation factor IX |
| FAM20A | family with sequence similarity 20, member A |
| FAM20B | family with sequence similarity 20, member B |
| FAM20C | family with sequence similarity 20, member C |
| HABP2 | hyaluronan binding protein 2 |
| HMSD | histocompatibility (minor) serpin domain containing |
| HPSE | heparanase |
| HPSE2 | heparanase 2 |
| HRG | histidine-rich glycoprotein |
| HTRA1 | HtrA serine peptidase 1 |
| HTRA3 | HtrA serine peptidase 3 |
| HTRA4 | HtrA serine peptidase 4 |
| HYAL1 | hyaluronoglucosaminidase 1 |
| HYAL2 | hyaluronoglucosaminidase 2 |
| HYAL3 | hyaluronoglucosaminidase 3 |
| HYAL4 | hyaluronoglucosaminidase 4 |
| ITIH1 | inter-alpha (globulin) inhibitor H1 |
| ITIH2 | inter-alpha (globulin) inhibitor H2 |
| ITIH3 | inter-alpha (globulin) inhibitor H3 |
| ITIH4 | inter-alpha (globulin) inhibitor H4 (plasma Kallikrein-sensitive glycoprotein) |
| ITIH5 | inter-alpha (globulin) inhibitor H5 |
| ITIH6 | inter-alpha (globulin) inhibitor H5-like |
| KAZALD1 | Kazal-type serine peptidase inhibitor domain 1 |
| KNG1 | kininogen 1 |
| KY | kyphoscoliosis peptidase |
| LEPRE1 | leucine proline-enriched proteoglycan (leprecan) 1 |
| LEPREL1 | leprecan-like 1 |
| LEPREL2 | leprecan-like 2 |
| LOX | lysyl oxidase |
| LOXL1 | lysyl oxidase-like 1 |
| LOXL2 | lysyl oxidase-like 2 |
| LOXL3 | lysyl oxidase-like 3 |
| LOXL4 | lysyl oxidase-like 4 |
| LPA | lipoprotein, Lp(a) |
| MASP1 | mannan-binding lectin serine peptidase 1 (C4/C2 activating component of Ra-reactive factor) |
| MASP2 | mannan-binding lectin serine peptidase 2 |
| MEP1A | meprin A, alpha (PABA peptide hydrolase) |
| MEP1B | meprin A, beta |
| MMP1 | matrix metallopeptidase 1 (interstitial collagenase) |
| MMP10 | matrix metallopeptidase 10 (stromelysin 2) |
| MMP11 | matrix metallopeptidase 11 (stromelysin 3) |
| MMP12 | matrix metallopeptidase 12 (macrophage elastase) |
| MMP13 | matrix metallopeptidase 13 (collagenase 3) |
| MMP14 | matrix metallopeptidase 14 (membrane-inserted) |
| MMP15 | matrix metallopeptidase 15 (membrane-inserted) |
| MMP16 | matrix metallopeptidase 16 (membrane-inserted) |

| | |
|---|---|
| MMP17 | matrix metallopeptidase 17 (membrane-inserted) |
| MMP19 | matrix metallopeptidase 19 |
| MMP2 | matrix metallopeptidase 2 (gelatinase A, 72kDa gelatinase, 72kDa type IV collagenase) |
| MMP20 | matrix metallopeptidase 20 |
| MMP21 | matrix metallopeptidase 21 |
| MMP23B | matrix metallopeptidase 23B |
| MMP24 | matrix metallopeptidase 24 (membrane-inserted) |
| MMP25 | matrix metallopeptidase 25 |
| MMP26 | matrix metallopeptidase 26 |
| MMP27 | matrix metallopeptidase 27 |
| MMP28 | matrix metallopeptidase 28 |
| MMP3 | matrix metallopeptidase 3 (stromelysin 1, progelatinase) |
| MMP7 | matrix metallopeptidase 7 (matrilysin, uterine) |
| MMP8 | matrix metallopeptidase 8 (neutrophil collagenase) |
| MMP9 | matrix metallopeptidase 9 (gelatinase B, 92kDa gelatinase, 92kDa type IV collagenase) |
| NGLY1 | N-glycanase 1 |
| OGFOD1 | 2-oxoglutarate and iron-dependent oxygenase domain containing 1 |
| OGFOD2 | 2-oxoglutarate and iron-dependent oxygenase domain containing 2 |
| P4HA1 | prolyl 4-hydroxylase, alpha polypeptide I |
| P4HA2 | prolyl 4-hydroxylase, alpha polypeptide II |
| P4HA3 | prolyl 4-hydroxylase, alpha polypeptide III |
| P4HTM | prolyl 4-hydroxylase, transmembrane (endoplasmic reticulum) |
| PAMR1 | peptidase domain containing associated with muscle regeneration 1 |
| PAPPA | pregnancy-associated plasma protein A, pappalysin 1 |
| PAPPA2 | pappalysin 2 |
| PCSK5 | proprotein convertase subtilisin/kexin type 5 |
| PCSK6 | proprotein convertase subtilisin/kexin type 6 |
| PI3 | peptidase inhibitor 3, skin-derived |
| PLAT | plasminogen activator, tissue |
| PLAU | plasminogen activator, urokinase |
| PLG | plasminogen |
| PLOD1 | procollagen-lysine 1, 2-oxoglutarate 5-dioxygenase 1 |
| PLOD2 | procollagen-lysine, 2-oxoglutarate 5-dioxygenase 2 |
| PLOD3 | procollagen-lysine, 2-oxoglutarate 5-dioxygenase 3 |
| PRSS1 | protease, serine, 1 (trypsin 1) |
| PRSS12 | protease, serine, 12 (neurotrypsin, motopsin) |
| PRSS2 | protease, serine, 2 (trypsin 2) |
| PRSS3 | protease, serine, 3 |
| PZP | pregnancy-zone protein |
| SERPINA1 | serpin peptidase inhibitor, clade A (alpha-1 antiproteinase, antitrypsin), member 1 |
| SERPINA10 | serpin peptidase inhibitor, clade A (alpha-1 antiproteinase, antitrypsin), member 10 |
| SERPINA11 | serpin peptidase inhibitor, clade A (alpha-1 antiproteinase, antitrypsin), member 11 |
| SERPINA12 | serpin peptidase inhibitor, clade A (alpha-1 antiproteinase, antitrypsin), member 12 |
| SERPINA2 | serpin peptidase inhibitor, clade A (alpha-1 antiproteinase, antitrypsin), member 2 |

| | |
|---|---|
| SERPINA3 | serpin peptidase inhibitor, clade A (alpha-1 antiproteinase, antitrypsin), member 3 |
| SERPINA4 | serpin peptidase inhibitor, clade A (alpha-1 antiproteinase, antitrypsin), member 4 |
| SERPINA5 | serpin peptidase inhibitor, clade A (alpha-1 antiproteinase, antitrypsin), member 5 |
| SERPINA6 | serpin peptidase inhibitor, clade A (alpha-1 antiproteinase, antitrypsin), member 6 |
| SERPINA7 | serpin peptidase inhibitor, clade A (alpha-1 antiproteinase, antitrypsin), member 7 |
| SERPINA9 | serpin peptidase inhibitor, clade A (alpha-1 antiproteinase, antitrypsin), member 9 |
| SERPINB1 | serpin peptidase inhibitor, clade B (ovalbumin), member 1 |
| SERPINB10 | serpin peptidase inhibitor, clade B (ovalbumin), member 10 |
| SERPINB11 | serpin peptidase inhibitor, clade B (ovalbumin), member 11 (gene/pseudogene) |
| SERPINB12 | serpin peptidase inhibitor, clade B (ovalbumin), member 12 |
| SERPINB13 | serpin peptidase inhibitor, clade B (ovalbumin), member 13 |
| SERPINB2 | serpin peptidase inhibitor, clade B (ovalbumin), member 2 |
| SERPINB3 | serpin peptidase inhibitor, clade B (ovalbumin), member 3 |
| SERPINB4 | serpin peptidase inhibitor, clade B (ovalbumin), member 4 |
| SERPINB5 | serpin peptidase inhibitor, clade B (ovalbumin), member 5 |
| SERPINB6 | serpin peptidase inhibitor, clade B (ovalbumin), member 6 |
| SERPINB7 | serpin peptidase inhibitor, clade B (ovalbumin), member 7 |
| SERPINB8 | serpin peptidase inhibitor, clade B (ovalbumin), member 8 |
| SERPINB9 | serpin peptidase inhibitor, clade B (ovalbumin), member 9 |
| SERPINC1 | serpin peptidase inhibitor, clade C (antithrombin), member 1 |
| SERPIND1 | serpin peptidase inhibitor, clade D (heparin cofactor), member 1 |
| SERPINE1 | serpin peptidase inhibitor, clade E (nexin, plasminogen activator inhibitor type 1), member 1 |
| SERPINE2 | serpin peptidase inhibitor, clade E (nexin, plasminogen activator inhibitor type 1), member 2 |
| SERPINE3 | serpin peptidase inhibitor, clade E (nexin, plasminogen activator inhibitor type 1), member 3 |
| SERPINF1 | serpin peptidase inhibitor, clade F (alpha-2 antiplasmin, pigment epithelium derived factor), member 1 |
| SERPINF2 | serpin peptidase inhibitor, clade F (alpha-2 antiplasmin, pigment epithelium derived factor), member 2 |
| SERPING1 | serpin peptidase inhibitor, clade G (C1 inhibitor), member 1 |
| SERPINH1 | serpin peptidase inhibitor, clade H (heat shock protein 47), member 1, (collagen binding protein 1) |
| SERPINI1 | serpin peptidase inhibitor, clade I (neuroserpin), member 1 |
| SERPINI2 | serpin peptidase inhibitor, clade I (pancpin), member 2 |
| SLPI | secretory leukocyte peptidase inhibitor |
| SPAM1 | sperm adhesion molecule 1 (PH-20 hyaluronidase, zona pellucida binding) |
| ST14 | suppression of tumorigenicity 14 (colon carcinoma) |
| SULF1 | sulfatase 1 |
| SULF2 | sulfatase 2 |
| TGM1 | transglutaminase 1 (K polypeptide epidermal type I, protein-glutamine-gamma-glutamyltransferase) |

| | |
|---|---|
| TGM2 | transglutaminase 2 (C polypeptide, protein-glutamine-gamma-glutamyltransferase) |
| TGM3 | transglutaminase 3 (E polypeptide, protein-glutamine-gamma-glutamyltransferase) |
| TGM4 | transglutaminase 4 (prostate) |
| TGM5 | transglutaminase 5 |
| TGM6 | transglutaminase 6 |
| TGM7 | transglutaminase 7 |
| TIMP1 | TIMP metallopeptidase inhibitor 1 |
| TIMP2 | TIMP metallopeptidase inhibitor 2 |
| TIMP3 | TIMP metallopeptidase inhibitor 3 |
| TIMP4 | TIMP metallopeptidase inhibitor 4 |
| TLL1 | tolloid-like 1 |
| TLL2 | tolloid-like 2 |
| TMPRSS15 | protease, serine, 7 (enterokinase) |
| AMH | anti-Mullerian hormone |
| ANGPT1 | angiopoietin 1 |
| ANGPT2 | angiopoietin 2 |
| ANGPT4 | angiopoietin 4 |
| ANGPTL1 | angiopoietin-like 1 |
| ANGPTL2 | angiopoietin-like 2 |
| ANGPTL3 | angiopoietin-like 3 |
| ANGPTL4 | angiopoietin-like 4 |
| ANGPTL5 | angiopoietin-like 5 |
| ANGPTL6 | angiopoietin-like 6 |
| ANGPTL7 | angiopoietin-like 7 |
| AREG | amphiregulin |
| ARTN | artemin |
| BDNF | brain-derived neurotrophic factor |
| BMP10 | bone morphogenetic protein 10 |
| BMP15 | bone morphogenetic protein 15 |
| BMP2 | bone morphogenetic protein 2 |
| BMP3 | bone morphogenetic protein 3 |
| BMP4 | bone morphogenetic protein 4 |
| BMP5 | bone morphogenetic protein 5 |
| BMP6 | bone morphogenetic protein 6 |
| BMP7 | bone morphogenetic protein 7 |
| BMP8A | bone morphogenetic protein 8a |
| BMP8B | bone morphogenetic protein 8b |
| BRINP2 | family with sequence similarity 5, member B |
| BRINP3 | family with sequence similarity 5, member C |
| BTC | betacellulin |
| C1QTNF9B | C1q and tumor necrosis factor related protein 9B |
| CBLN1 | cerebellin 1 precursor |
| CBLN2 | cerebellin 2 precursor |
| CBLN3 | cerebellin 3 precursor |
| CBLN4 | cerebellin 4 precursor |
| CCBE1 | collagen and calcium binding EGF domains 1 |
| CCL1 | chemokine (C-C motif) ligand 1 |
| CCL11 | chemokine (C-C motif) ligand 11 |
| CCL13 | chemokine (C-C motif) ligand 13 |
| CCL14 | chemokine (C-C motif) ligand 14 |

| | |
|---|---|
| CCL15 | chemokine (C-C motif) ligand 15 |
| CCL16 | chemokine (C-C motif) ligand 16 |
| CCL17 | chemokine (C-C motif) ligand 17 |
| CCL18 | chemokine (C-C motif) ligand 18 (pulmonary and activation-regulated) |
| CCL19 | chemokine (C-C motif) ligand 19 |
| CCL2 | chemokine (C-C motif) ligand 2 |
| CCL20 | chemokine (C-C motif) ligand 20 |
| CCL21 | chemokine (C-C motif) ligand 21 |
| CCL22 | chemokine (C-C motif) ligand 22 |
| CCL23 | chemokine (C-C motif) ligand 23 |
| CCL24 | chemokine (C-C motif) ligand 24 |
| CCL25 | chemokine (C-C motif) ligand 25 |
| CCL26 | chemokine (C-C motif) ligand 26 |
| CCL27 | chemokine (C-C motif) ligand 27 |
| CCL28 | chemokine (C-C motif) ligand 28 |
| CCL3 | chemokine (C-C motif) ligand 3 |
| CCL3L3 | chemokine (C-C motif) ligand 3-like 3 |
| CCL4 | chemokine (C-C motif) ligand 4 |
| CCL4L1 | chemokine (C-C motif) ligand 4-like 1 |
| CCL4L2 | chemokine (C-C motif) ligand 4-like 2 |
| CCL5 | chemokine (C-C motif) ligand 5 |
| CCL7 | chemokine (C-C motif) ligand 7 |
| CCL8 | chemokine (C-C motif) ligand 8 |
| CFC1 | cripto, FRL-1, cryptic family 1 |
| CFC1B | cripto, FRL-1, cryptic family 1B |
| CHRD | chordin |
| CHRDL1 | chordin-like 1 |
| CHRDL2 | chordin-like 2 |
| CLCF1 | cardiotrophin-like cytokine factor 1 |
| CNTF | ciliary neurotrophic factor |
| CRHBP | corticotropin releasing hormone binding protein |
| CRLF1 | cytokine receptor-like factor 1 |
| CRLF3 | cytokine receptor-like factor 3 |
| CRNN | cornulin |
| CSF1 | colony stimulating factor 1 (macrophage) |
| CSF2 | colony stimulating factor 2 (granulocyte-macrophage) |
| CSF3 | colony stimulating factor 3 (granulocyte) |
| CSH1 | chorionic somatomammotropin hormone 1 (placental lactogen) |
| CSH2 | chorionic somatomammotropin hormone 2 |
| CSHL1 | chorionic somatomammotropin hormone-like 1 |
| CTF1 | cardiotrophin 1 |
| CX3CL1 | chemokine (C-X3-C motif) ligand 1 |
| CXCL1 | chemokine (C-X-C motif) ligand 1 (melanoma growth stimulating activity, alpha) |
| CXCL10 | chemokine (C-X-C motif) ligand 10 |
| CXCL11 | chemokine (C-X-C motif) ligand 11 |
| CXCL12 | chemokine (C-X-C motif) ligand 12 (stromal cell-derived factor 1) |
| CXCL13 | chemokine (C-X-C motif) ligand 13 |
| CXCL14 | chemokine (C-X-C motif) ligand 14 |
| CXCL2 | chemokine (C-X-C motif) ligand 2 |
| CXCL3 | chemokine (C-X-C motif) ligand 3 |
| CXCL5 | chemokine (C-X-C motif) ligand 5 |

| | |
|---|---|
| CXCL6 | chemokine (C-X-C motif) ligand 6 (granulocyte chemotactic protein 2) |
| CXCL8 | interleukin 8 |
| CXCL9 | chemokine (C-X-C motif) ligand 9 |
| DHH | desert hedgehog homolog (Drosophila) |
| EBI3 | Epstein-Barr virus induced 3 |
| EDA | ectodysplasin A |
| EGF | epidermal growth factor (beta-urogastrone) |
| EGFL6 | EGF-like-domain, multiple 6 |
| EGFL7 | EGF-like-domain, multiple 7 |
| EGFL8 | EGF-like-domain, multiple 8 |
| EPGN | epithelial mitogen homolog (mouse) |
| EPO | erythropoietin |
| EREG | epiregulin |
| FASLG | Fas ligand (TNF superfamily, member 6) |
| FGF1 | fibroblast growth factor 1 (acidic) |
| FGF10 | fibroblast growth factor 10 |
| FGF11 | fibroblast growth factor 11 |
| FGF12 | fibroblast growth factor 12 |
| FGF13 | fibroblast growth factor 13 |
| FGF14 | fibroblast growth factor 14 |
| FGF16 | fibroblast growth factor 16 |
| FGF17 | fibroblast growth factor 17 |
| FGF18 | fibroblast growth factor 18 |
| FGF19 | fibroblast growth factor 19 |
| FGF2 | fibroblast growth factor 2 (basic) |
| FGF20 | fibroblast growth factor 20 |
| FGF21 | fibroblast growth factor 21 |
| FGF22 | fibroblast growth factor 22 |
| FGF23 | fibroblast growth factor 23 |
| FGF3 | fibroblast growth factor 3 (murine mammary tumor virus integration site (v-int-2) oncogene homolog) |
| FGF4 | fibroblast growth factor 4 |
| FGF5 | fibroblast growth factor 5 |
| FGF6 | fibroblast growth factor 6 |
| FGF7 | fibroblast growth factor 7 (keratinocyte growth factor) |
| FGF8 | fibroblast growth factor 8 (androgen-induced) |
| FGF9 | fibroblast growth factor 9 (glia-activating factor) |
| FGFBP1 | fibroblast growth factor binding protein 1 |
| FGFBP2 | fibroblast growth factor binding protein 2 |
| FGFBP3 | fibroblast growth factor binding protein 3 |
| FIGF | c-fos induced growth factor (vascular endothelial growth factor D) |
| FLG | filaggrin |
| FLG2 | filaggrin family member 2 |
| FLT3LG | fms-related tyrosine kinase 3 ligand |
| FRZB | frizzled-related protein |
| FST | follistatin |
| FSTL1 | follistatin-like 1 |
| FSTL3 | follistatin-like 3 (secreted glycoprotein) |
| GDF1 | growth differentiation factor 1 |
| GDF10 | growth differentiation factor 10 |
| GDF11 | growth differentiation factor 11 |
| GDF15 | growth differentiation factor 15 |

| | |
|---|---|
| GDF2 | growth differentiation factor 2 |
| GDF3 | growth differentiation factor 3 |
| GDF5 | growth differentiation factor 5 |
| GDF6 | growth differentiation factor 6 |
| GDF7 | growth differentiation factor 7 |
| GDF9 | growth differentiation factor 9 |
| GDNF | glial cell derived neurotrophic factor |
| GH1 | growth hormone 1 |
| GH2 | growth hormone 2 |
| HBEGF | heparin-binding EGF-like growth factor |
| HCFC1 | host cell factor C1 (VP16-accessory protein) |
| HCFC2 | host cell factor C2 |
| HGF | hepatocyte growth factor (hepapoietin A; scatter factor) |
| HGFAC | HGF activator |
| HHIP | hedgehog interacting protein |
| HRNR | hornerin |
| IFNA1 | interferon, alpha 1 |
| IFNA10 | interferon, alpha 10 |
| IFNA13 | interferon, alpha 13 |
| IFNA14 | interferon, alpha 14 |
| IFNA16 | interferon, alpha 16 |
| IFNA17 | interferon, alpha 17 |
| IFNA2 | interferon, alpha 2 |
| IFNA21 | interferon, alpha 21 |
| IFNA4 | interferon, alpha 4 |
| IFNA5 | interferon, alpha 5 |
| IFNA6 | interferon, alpha 6 |
| IFNA7 | interferon, alpha 7 |
| IFNA8 | interferon, alpha 8 |
| IFNB1 | interferon, beta 1, fibroblast |
| IFNE | interferon, epsilon |
| IFNG | interferon, gamma |
| IFNK | interferon, kappa |
| IFNW1 | interferon, omega 1 |
| IGF1 | insulin-like growth factor 1 (somatomedin C) |
| IGF2 | insulin-like growth factor 2 (somatomedin A) |
| IHH | Indian hedgehog homolog (Drosophila) |
| IL10 | interleukin 10 |
| IL11 | interleukin 11 |
| IL12A | interleukin 12A (natural killer cell stimulatory factor 1, cytotoxic lymphocyte maturation factor 1, p35) |
| IL12B | interleukin 12B (natural killer cell stimulatory factor 2, cytotoxic lymphocyte maturation factor 2, p40) |
| IL13 | interleukin 13 |
| IL15 | interleukin 15 |
| IL16 | interleukin 16 (lymphocyte chemoattractant factor) |
| IL17A | interleukin 17A |
| IL17B | interleukin 17B |
| IL17C | interleukin 17C |
| IL17D | interleukin 17D |
| IL17F | interleukin 17F |
| IL18 | interleukin 18 (interferon-gamma-inducing factor) |

| | |
|---|---|
| IL19 | interleukin 19 |
| IL1A | interleukin 1, alpha |
| IL1B | interleukin 1, beta |
| IL1F10 | interleukin 1 family, member 10 (theta) |
| IL1F5 | interleukin 1 family, member 5 (delta) |
| IL1F6 | interleukin 1 family, member 6 (epsilon) |
| IL1F7 | interleukin 1 family, member 7 (zeta) |
| IL1F8 | interleukin 1 family, member 8 (eta) |
| IL1F9 | interleukin 1 family, member 9 |
| IL1RN | interleukin 1 receptor antagonist |
| IL2 | interleukin 2 |
| IL20 | interleukin 20 |
| IL22 | interleukin 22 |
| IL23A | interleukin 23, alpha subunit p19 |
| IL24 | interleukin 24 |
| IL25 | interleukin 25 |
| IL26 | interleukin 26 |
| IL3 | interleukin 3 (colony-stimulating factor, multiple) |
| IL34 | interleukin 34 |
| IL4 | interleukin 4 |
| IL5 | interleukin 5 (colony-stimulating factor, eosinophil) |
| IL6 | interleukin 6 (interferon, beta 2) |
| IL7 | interleukin 7 |
| IL9 | interleukin 9 |
| INHA | inhibin, alpha |
| INHBA | inhibin, beta A |
| INHBB | inhibin, beta B |
| INHBC | inhibin, beta C |
| INHBE | inhibin, beta E |
| INS | insulin |
| INS-IGF2 | INS-IGF2 readthrough transcript |
| INSL3 | insulin-like 3 (Leydig cell) |
| INSL5 | insulin-like 5 |
| INSL6 | insulin-like 6 |
| ISM1 | isthmin 1 homolog (zebrafish) |
| ISM2 | isthmin 2 homolog (zebrafish) |
| KITLG | KIT ligand |
| LEFTY1 | left-right determination factor 1 |
| LEFTY2 | left-right determination factor 2 |
| LEP | leptin |
| LIF | leukemia inhibitory factor (cholinergic differentiation factor) |
| LTA | lymphotoxin alpha (TNF superfamily, member 1) |
| LTB | lymphotoxin beta (TNF superfamily, member 3) |
| MDK | midkine (neurite growth-promoting factor 2) |
| MEGF10 | multiple EGF-like-domains 10 |
| MEGF11 | multiple EGF-like-domains 11 |
| MEGF6 | multiple EGF-like-domains 6 |
| MEGF8 | multiple EGF-like-domains 8 |
| MEGF9 | multiple EGF-like-domains 9 |
| MST1 | macrophage stimulating 1 (hepatocyte growth factor-like) |
| MST1L | macrophage stimulating 1 (hepatocyte growth factor-like) pseudogene 9 |

| | |
|---|---|
| MSTN | myostatin |
| NGF | nerve growth factor (beta polypeptide) |
| NODAL | nodal homolog (mouse) |
| NRG1 | neuregulin 1 |
| NRG2 | neuregulin 2 |
| NRG3 | neuregulin 3 |
| NRG4 | neuregulin 4 |
| NRTN | neurturin |
| NTF3 | neurotrophin 3 |
| NTF4 | neurotrophin 4 |
| OSM | oncostatin M |
| PDGFA | platelet-derived growth factor alpha polypeptide |
| PDGFB | platelet-derived growth factor beta polypeptide (simian sarcoma viral (v-sis) oncogene homolog) |
| PDGFC | platelet derived growth factor C |
| PDGFD | platelet derived growth factor D |
| PF4 | platelet factor 4 |
| PF4V1 | platelet factor 4 variant 1 |
| PGF | placental growth factor |
| PIK3IP1 | phosphoinositide-3-kinase interacting protein 1 |
| PPBP | pro-platelet basic protein (chemokine (C-X-C motif) ligand 7) |
| PRL | prolactin |
| PSPN | persephin |
| PTN | pleiotrophin |
| RPTN | repetin |
| S100A1 | S100 calcium binding protein A1 |
| S100A10 | S100 calcium binding protein A10 |
| S100A11 | S100 calcium binding protein A11 |
| S100A12 | S100 calcium binding protein A12 |
| S100A13 | S100 calcium binding protein A13 |
| S100A14 | S100 calcium binding protein A14 |
| S100A16 | S100 calcium binding protein A16 |
| S100A2 | S100 calcium binding protein A2 |
| S100A3 | S100 calcium binding protein A3 |
| S100A4 | S100 calcium binding protein A4 |
| S100A5 | S100 calcium binding protein A5 |
| S100A6 | S100 calcium binding protein A6 |
| S100A7 | S100 calcium binding protein A7 |
| S100A7A | S100 calcium binding protein A7A |
| S100A7L2 | S100 calcium binding protein A7-like 2 |
| S100A8 | S100 calcium binding protein A8 |
| S100A9 | S100 calcium binding protein A9 |
| S100B | S100 calcium binding protein B |
| S100G | S100 calcium binding protein G |
| S100P | S100 calcium binding protein P |
| S100Z | S100 calcium binding protein Z |
| SCUBE1 | signal peptide, CUB domain, EGF-like 1 |
| SCUBE2 | signal peptide, CUB domain, EGF-like 2 |
| SCUBE3 | signal peptide, CUB domain, EGF-like 3 |
| SFRP1 | secreted frizzled-related protein 1 |
| SFRP2 | secreted frizzled-related protein 2 |
| SFRP4 | secreted frizzled-related protein 4 |

| | |
|---|---|
| SFRP5 | secreted frizzled-related protein 5 |
| SHH | sonic hedgehog homolog (Drosophila) |
| TCHH | trichohyalin |
| TCHHL1 | trichohyalin-like 1 |
| TDGF1 | teratocarcinoma-derived growth factor 1 |
| TGFA | transforming growth factor, alpha |
| TGFB1 | transforming growth factor, beta 1 |
| TGFB2 | transforming growth factor, beta 2 |
| TGFB3 | transforming growth factor, beta 3 |
| THPO | thrombopoietin |
| TNF | tumor necrosis factor (TNF superfamily, member 2) |
| TNFSF10 | tumor necrosis factor (ligand) superfamily, member 10 |
| TNFSF11 | tumor necrosis factor (ligand) superfamily, member 11 |
| TNFSF12 | tumor necrosis factor (ligand) superfamily, member 12 |
| TNFSF13 | tumor necrosis factor (ligand) superfamily, member 13 |
| TNFSF13B | tumor necrosis factor (ligand) superfamily, member 13b |
| TNFSF14 | tumor necrosis factor (ligand) superfamily, member 14 |
| TNFSF15 | tumor necrosis factor (ligand) superfamily, member 15 |
| TNFSF18 | tumor necrosis factor (ligand) superfamily, member 18 |
| TNFSF4 | tumor necrosis factor (ligand) superfamily, member 4 |
| TNFSF8 | tumor necrosis factor (ligand) superfamily, member 8 |
| TNFSF9 | tumor necrosis factor (ligand) superfamily, member 9 |
| TPO | thyroid peroxidase |
| VEGFA | vascular endothelial growth factor A |
| VEGFB | vascular endothelial growth factor B |
| VEGFC | vascular endothelial growth factor C |
| VWC2 | von Willebrand factor C domain containing 2 |
| VWC2L | von Willebrand factor C domain-containing protein 2-like |
| WFIKKN1 | WAP, follistatin/kazal, immunoglobulin, kunitz and netrin domain containing 1 |
| WFIKKN2 | WAP, follistatin/kazal, immunoglobulin, kunitz and netrin domain containing 2 |
| WIF1 | WNT inhibitory factor 1 |
| WNT1 | wingless-type MMTV integration site family, member 1 |
| WNT10A | wingless-type MMTV integration site family, member 10A |
| WNT10B | wingless-type MMTV integration site family, member 10B |
| WNT11 | wingless-type MMTV integration site family, member 11 |
| WNT16 | wingless-type MMTV integration site family, member 16 |
| WNT2 | wingless-type MMTV integration site family member 2 |
| WNT2B | wingless-type MMTV integration site family, member 2B |
| WNT3 | wingless-type MMTV integration site family, member 3 |
| WNT3A | wingless-type MMTV integration site family, member 3A |
| WNT4 | wingless-type MMTV integration site family, member 4 |
| WNT5A | wingless-type MMTV integration site family, member 5A |
| WNT5B | wingless-type MMTV integration site family, member 5B |
| WNT6 | wingless-type MMTV integration site family, member 6 |
| WNT7A | wingless-type MMTV integration site family, member 7A |
| WNT7B | wingless-type MMTV integration site family, member 7B |
| WNT8A | wingless-type MMTV integration site family, member 8A |
| WNT8B | wingless-type MMTV integration site family, member 8B |
| WNT9A | wingless-type MMTV integration site family, member 9A |
| WNT9B | wingless-type MMTV integration site family, member 9B |

| | |
|---|---|
| XCL1 | chemokine (C motif) ligand 1 |
| XCL2 | chemokine (C motif) ligand 2 |
| ZFP91 | zinc finger protein 91 homolog (mouse) |
| ADAM1A | ADAM metallopeptidase domain 1, pseudogene |
| ADAM21P1 | ADAM metallopeptidase domain 21 pseudogene 1 |
| ADAM3A | ADAM metallopeptidase domain 3A (cyritestin 1) |
| ADAM3B | ADAM metallopeptidase domain 3B (non-functional) |
| ADAM5 | ADAM metallopeptidase domain 5, pseudogene |
| ADAM6 | ADAM metallopeptidase domain 6 (pseudogene) |
| ANXA2P2 | annexin A2 pseudogene 2 |
| ANXA8L2 | annexin A8-like 2 |
| BPIFA4P | breast cancer and salivary gland expression gene |
| C17orf101 | chromosome 17 open reading frame 101 |
| COL6A4P1 | collagen, type VI, alpha 4 pseudogene 1 |
| COL6A4P2 | collagen, type VI, alpha 4 pseudogene 2 |
| CST9L2 | Putative cystatin-9-like 2 |
| CTF2P | cardiotrophin 2, pseudogene |
| CTSL3P | cathepsin L family member 3 |
| CTSLP3 | similar to Cathepsin L1 |
| CTSLP6 | cathepsin L-like 6 |
| CTSLP7 | similar to Putative cathepsin L-like protein 6 |
| DMBT1P1 | deleted in malignant brain tumors 1 pseudogene |
| EGFEM1P | chromosome 3 open reading frame 50 |
| FGF7P2 | fibroblast growth factor 7 pseudogene 2 |
| gene_A6NLB4 | |
| HYALP1 | hyaluronoglucosaminidase pseudogene 1 |
| KGFLP1 | keratinocyte growth factor-like protein 1 |
| KGFLP2 | keratinocyte growth factor-like protein 2 |
| LOC400696 | lectin, galactoside-binding, soluble, 14-like |
| LOC728715 | similar to hCG38149 |
| LPAL2 | lipoprotein, Lp(a)-like 2 pseudogene |
| MBL1P | mannose-binding lectin (protein A) 1, pseudogene |
| MST1P2 | macrophage stimulating 1 (hepatocyte growth factor-like) pseudogene 2 |
| NCRNA00083 | non-protein coding RNA 83 |
| NEPNP | nephrocan, pseudogene |
| NTF6B | neurotrophin 6 beta (pseudogene) |
| OVOS1 | UP:Ovostatin 1 |
| OVOS2 | UP:Ovostatin 2 |
| PPBPP1 | pro-platelet basic protein-like 1 |
| PRSS3P1 | trypsinogen B |
| SERPINA13P | serpin peptidase inhibitor, clade A (alpha-1 antiproteinase, antitrypsin), member 13 (pseudogene) |
| TNFSF12-TNFSF13 | TNFSF12-TNFSF13 readthrough |
| TNXA | tenascin XA pseudogene |
| UNQ5830 | AILT5830 |

Table S2 Drug-gene interactions

| gene | drug |
|---|---|
| TG | GENISTEIN |
| TG | CALCITRIOL |
| TG | AMINOGLUTETHIMIDE |
| TG | AMIODARONE |
| TG | ALCOHOL |
| TG | OCTREOTIDE |
| TG | ACEBUTOLOL |
| TG | HEXAMETHYLENEBISACETAMIDE |
| TG | ALDESLEUKIN |
| TG | PHENYTOIN |
| TG | PREDNISONE |
| TG | DEXAMETHASONE |
| TG | ISOTRETINOIN |
| TG | PREDNISOLONE |
| TG | AZACITIDINE |
| TG | RETINOL |
| TG | ROSIGLITAZONE |
| TG | FLUDEOXYGLUCOSE-F18 |
| TP53 | BORTEZOMIB |
| TP53 | ALEXIDINE DIHYDROCHLORIDE |
| TP53 | E-FLUPENTIXOL |
| TP53 | DOXORUBICIN |
| TP53 | N6-BENZYLADENOSINE |
| TP53 | CLOFOCTOL |
| TP53 | TAMOXIFEN CITRATE |
| TP53 | DOPAMINE |
| TP53 | EMODIN |
| TP53 | MERCAPTOPURINE |
| TP53 | CHEMBL261123 |
| TP53 | (Z)-PIPLARTINE |
| TP53 | MICHLER'S KETONE |
| TP53 | CAPIVASERTIB |
| TP53 | FENRETINIDE |
| TP53 | METHIMAZOLE |
| TP53 | RG-7602 |
| TP53 | DAUNORUBICIN HYDROCHLORIDE |
| TP53 | PAROXETINE HYDROCHLORIDE |
| TP53 | RHODOMYRTOXIN B |
| TP53 | CHEMBL261118 |
| TP53 | ZIRAM |
| TP53 | DAIDZEIN |
| TP53 | CHEMBL1241268 |
| TP53 | CHLORPROMAZINE |
| TP53 | ENALAPRIL |
| TP53 | CHEMBL578890 |
| TP53 | TRIFLOXYSTROBIN |
| TP53 | LOPERAMIDE |
| TP53 | CHEMBL261693 |
| TP53 | O-DEMETHYLATED ADAPALENE |
| TP53 | MELPHALAN |
| TP53 | METHYL ROBUSTONE |
| TP53 | CHEMBL265686 |
| TP53 | AVASTIN |
| TP53 | TEMOZOLOMIDE |
| TP53 | FURAZOLIDONE |

| | |
|---|---|
| TP53 | TANESPIMYCIN |
| TP53 | DAUNORUBICIN |
| TP53 | SERDEMETAN |
| TP53 | CHEMBL1702181 |
| TP53 | KINETIN RIBOSIDE |
| TP53 | CHEMBL1397308 |
| TP53 | CHEMBL528807 |
| TP53 | DINOSEB |
| TP53 | CHEMBL405914 |
| TP53 | CHEMBL1169388 |
| TP53 | CYAZOFAMID |
| TP53 | CHEMBL422942 |
| TP53 | CHEMBL16685 |
| TP53 | TPCK |
| TP53 | IOXYNIL |
| TP53 | CHEMBL405317 |
| TP53 | ALT-801 |
| TP53 | ATRACTYLENOLIDE I |
| TP53 | HOMIDIUM BROMIDE |
| TP53 | TEBUFENPYRAD |
| TP53 | ROTENONE |
| TP53 | STREPTONIGRIN |
| TP53 | CETUXIMAB |
| TP53 | CHEMBL578512 |
| TP53 | (R,S)-INDATRALINE |
| TP53 | PEMBROLIZUMAB |
| TP53 | GEMCITABINE |
| TP53 | CARBOPLATIN |
| TP53 | TRACAZOLATE |
| TP53 | CHEMBL533226 |
| TP53 | PLUMBAGIN |
| TP53 | IODOACETAMIDE |
| TP53 | CHEMBL271023 |
| TP53 | CHEMBL408653 |
| TP53 | BEVACIZUMAB |
| TP53 | BENZO(K)FLUORANTHENE |
| TP53 | INDOPROFEN |
| TP53 | BROXYQUINOLINE |
| TP53 | CARBENDAZIM |
| TP53 | CHEMBL267160 |
| TP53 | CHEMBL285819 |
| TP53 | MITOXANTRONE |
| TP53 | LY-3009120 |
| TP53 | GENISTEIN |
| TP53 | CHEMBL1214407 |
| TP53 | GARLIC |
| TP53 | CIS-RESVERATROL |
| TP53 | CHEMBL408850 |
| TP53 | BITHIONOL |
| TP53 | CHLOROTHALONIL |
| TP53 | PD-0325901 |
| TP53 | (S)APOMORPHINE |
| TP53 | CHEMBL177809 |
| TP53 | ABEMACICLIB |
| TP53 | IRINOTECAN |
| TP53 | RIBAVIRIN |

| | |
|---|---|
| TP53 | CHEMBL27006 |
| TP53 | CADMIUM DICHLORIDE |
| TP53 | HYDRALAZINE HYDROCHLORIDE |
| TP53 | CHEMBL579550 |
| TP53 | SALMETEROL XINAFOATE |
| TP53 | DIHYDROGAMBOGIC ACID |
| TP53 | CHLORAMBUCIL |
| TP53 | CHEMBL1346468 |
| TP53 | ACEQUINOCYL |
| TP53 | PIPLARTINE |
| TP53 | CLIOQUINOL |
| TP53 | METHYLENE BLUE |
| TP53 | CLOTRIMAZOLE |
| TP53 | MUNDULONE |
| TP53 | 4-ISOTHIOUREIDOBUTYRONITRILE |
| TP53 | PF-04217903 |
| TP53 | 4-METHOXYDALBERGIONE |
| TP53 | IODOQUINOL |
| TP53 | PIMOZIDE |
| TP53 | CHEMBL2002487 |
| TP53 | CHEMBL1333386 |
| TP53 | IBRUTINIB |
| TP53 | CHEMBL201325 |
| TP53 | 3',4' DICHLOROBENZAMIL |
| TP53 | QUINALIZARIN |
| TP53 | BENZETHONIUM CHLORIDE |
| TP53 | CHEMBL1343568 |
| TP53 | GRANISETRON |
| TP53 | NEBULARINE |
| TP53 | FANCHININ |
| TP53 | TRIFLUPROMAZINE |
| TP53 | CHEMBL259422 |
| TP53 | APOMORPHINE |
| TP53 | CHEMBL582473 |
| TP53 | CHEMBL88272 |
| TP53 | CHEMBL1801219 |
| TP53 | SAPANISERTIB |
| TP53 | CHEMBL184450 |
| TP53 | OLVANIL |
| TP53 | BENOMYL |
| TP53 | ALPHA-NAPHTHOFLAVONE |
| TP53 | DEMCIZUMAB |
| TP53 | CHEMBL421215 |
| TP53 | CHEMBL530049 |
| TP53 | OXALIPLATIN |
| TP53 | CHEMBL260213 |
| TP53 | KRESOXIM-METHYL |
| TP53 | OLEOYL DOPAMINE |
| TP53 | PROPYLTHIOURACIL |
| TP53 | FLUOROURACIL |
| TP53 | MERCURIC CHLORIDE |
| TP53 | CAPMATINIB |
| TP53 | CHEMBL599100 |
| TP53 | CGM-097 |
| TP53 | MITOXANTRONE HYDROCHLORIDE |
| TP53 | FLAVANONE |

| | |
|---|---|
| TP53 | PHENETHYLISOTHIOCYANATE |
| TP53 | CAULIBUGULONE D |
| TP53 | TRICLOCARBAN |
| TP53 | CHEMBL444376 |
| TP53 | CHEMBL600334 |
| TP53 | MECHLORETHAMINE HYDROCHLORIDE |
| TP53 | NICLOSAMIDE |
| TP53 | CHEMBL99408 |
| TP53 | PROPACHLOR |
| TP53 | ROBUSTIC ACID |
| TP53 | CHEMBL154580 |
| TP53 | CAPECITABINE |
| TP53 | FLUPHENAZINE |
| TP53 | DS-7423 |
| TP53 | PROPARGITE |
| TP53 | PACLITAXEL |
| TP53 | DIETHYLSTILBESTROL |
| TP53 | ENCORAFENIB |
| TP53 | CHEMBL85194 |
| TP53 | CLOFIBRATE |
| TP53 | CHEMBL591126 |
| TP53 | CHEMBL602150 |
| TP53 | NAVITOCLAX |
| TP53 | PROCHLORPERAZINE |
| TP53 | GALLIC ACID |
| TP53 | ABT 737 |
| TP53 | CHEMBL601757 |
| TP53 | CHEMBL601140 |
| TP53 | PAZOPANIB |
| TP53 | ETHOPROPAZINE HYDROCHLORIDE |
| TP53 | CHEMBL260560 |
| TP53 | CHEMBL260028 |
| TP53 | AZD-6482 |
| TP53 | RALOXIFENE |
| TP53 | RITUXIMAB |
| TP53 | GEDATOLISIB |
| TP53 | TRIAMTERENE |
| TP53 | 3-METHOXYCATECHOL |
| TP53 | ANTIMYCIN A |
| TP53 | CHEMBL411085 |
| TP53 | TRIFLURIDINE |
| TP53 | CHEMBL320361 |
| TP53 | GR-127935 |
| TP53 | PROGESTERONE |
| TP53 | CHEMBL259784 |
| TP53 | U-50488 METHANE SULFONATE |
| TP53 | DEOXIEPINEPHRINE |
| TP53 | CHEMBL260451 |
| TP53 | YONDELIS |
| TP53 | CHEMBL227959 |
| TP53 | CHEMBL600347 |
| TP53 | HALOPERIDOL |
| TP53 | PYRACLOSTROBIN |
| TP53 | POTASSIUM DICHROMATE |
| TP53 | PHENANTHROLINE |
| TP53 | THUNBERGINOL B |

| | |
|---|---|
| TP53 | CAULIBUGULONE C |
| TP53 | TIRAPAZAMINE |
| TP53 | BERZOSERTIB |
| TP53 | VORINOSTAT |
| TP53 | DACTOLISIB |
| TP53 | CHEMBL587620 |
| TP53 | CHEMBL590665 |
| TP53 | CHEMBL1522984 |
| TP53 | LB-100 |
| TP53 | CHEMBL563919 |
| TP53 | ONC-201 |
| TP53 | PYRITHIONE ZINC |
| TP53 | MITOMYCIN |
| TP53 | CHEMBL291143 |
| TP53 | DABRAFENIB |
| TP53 | RESVERATROL |
| TP53 | ETOPOSIDE |
| TP53 | METHOTREXATE |
| TP53 | CADMIUM ACETATE |
| TP53 | CHEMBL1389794 |
| TP53 | LAPACHONE |
| TP53 | CHEMBL528712 |
| TP53 | INDIRUBIN-3-MONOXIME |
| TP53 | (R)-INDOPROFEN |
| TP53 | CISPLATIN |
| TP53 | CHEMBL429095 |
| TP53 | PF-00477736 |
| TP53 | SANGUINARIUM |
| TP53 | DOXORUBICIN HYDROCHLORIDE |
| TP53 | LORLATINIB |
| TP53 | FUROXAN |
| TP53 | PRODIGIOSIN |
| TP53 | LONTUCIREV (REPLICATING ADENOVIRUS) |
| TP53 | ANISINDIONE |
| TP53 | CAMPTOSAR |
| TP53 | PROCHLORPERAZINE EDISYLATE |
| TP53 | PATULIN |
| TP53 | CHEMBL337173 |
| TP53 | CRIZOTINIB |
| TP53 | TERPYRIDINE |
| TP53 | 2-AMINOANTHRACENE |
| TP53 | SULCONAZOLE NITRATE |
| TP53 | CHEMBL26320 |
| TP53 | SANGUINARINE SULFATE |
| TP53 | CHEMBL66114 |
| TP53 | NIGULDIPINE |
| TP53 | CHEMBL545900 |
| TP53 | HEXACHLOROPHENE |
| TP53 | CHEMBL602922 |
| TP53 | VANOXERINE |
| TP53 | CHEMBL259805 |
| TP53 | SERTRALINE HYDROCHLORIDE |
| TP53 | APR-246 |
| TP53 | SELICICLIB |
| TP53 | METHYLPREDNISOLONE |
| TP53 | EMBELIN |

| | |
|---|---|
| TP53 | CHEMBL428789 |
| TP53 | CHEMBL591618 |
| TP53 | SIROLIMUS |
| TP53 | ELLIPTECINE |
| TP53 | EPIRUBICIN |
| TP53 | SERTRALINE |
| TP53 | CHELERYTHRINE |
| TP53 | DOCETAXEL |
| TP53 | CINNARIZINE |
| TP53 | CHLOROXINE |
| TP53 | CHEMBL35482 |
| TP53 | FENOFIBRATE |
| TP53 | ERLOTINIB |
| TP53 | TYRPHOSTIN A9 |
| TP53 | ONONIN |
| TP53 | SAR-405838 |
| TP53 | AZATHIOPRINE |
| TP53 | CHEMBL297784 |
| TP53 | RO-5045337 |
| TP53 | OLEAMIDE |
| TP53 | CLOMIPRAMINE |
| TP53 | GOSSYPOL |
| TP53 | AMOXAPINE |
| TP53 | FENTICLOR |
| TP53 | PANITUMUMAB |
| TP53 | AZD-7762 |
| TP53 | TRIACETYLRESVERATROL |
| TP53 | VEMURAFENIB |
| TP53 | CHLORANIL |
| TP53 | WARFARIN |
| TP53 | VENETOCLAX |
| TP53 | PHENYLMERCURIC ACETATE |
| TP53 | 7,8-DIHYDROXYFLAVONE |
| TP53 | THIMEROSAL |
| TP53 | CHEMBL233194 |
| TP53 | QUINACRINE DIHYDROCHLORIDE |
| TP53 | MANGOSTIN |
| TP53 | ECONAZOLE NITRATE |
| TP53 | DIMETHYL GAMBOGATE |
| TP53 | CHEMBL1326499 |
| TP53 | PHA-680632 |
| TP53 | RIMCAZOLE |
| TP53 | CHEMBL592123 |
| TP53 | CHEMBL580421 |
| TP53 | NORDIHYDROGUAIARETIC ACID |
| TP53 | CHEMBL596633 |
| TP53 | SB-224289 |
| TP53 | GANETESPIB |
| TP53 | CHEMBL259421 |
| TP53 | CHEMBL329673 |
| TP53 | DOXIL |
| TP53 | TEPRASIRAN |
| TP53 | CHEMBL242171 |
| TP53 | CHEMBL198759 |
| TP53 | CHEMBL1256697 |
| TP53 | CHEMBL599943 |

| | |
|---|---|
| TP53 | CHEMBL408563 |
| TP53 | CHEMBL406557 |
| TP53 | MAPROTILINE |
| TP53 | TEROXIRONE |
| TP53 | PIMASERTIB |
| TP53 | CHEMBL186526 |
| TP53 | CYTARABINE |
| TP53 | BENZALKONIUM CHLORIDE |
| TP53 | OLAPARIB |
| TP53 | 2,6-DIMETHOXYQUINONE |
| TP53 | NITAZOXANIDE |
| TP53 | NUTLIN-3 |
| TP53 | ALVESPIMYCIN |
| TP53 | CHEMBL410484 |
| TP53 | DUVELISIB |
| TP53 | TOPOTECAN |
| TP53 | PUROMYCIN |
| TP53 | CAULIBUGULONE B |
| TP53 | CHEMBL473735 |
| TP53 | CHEMBL259615 |
| TP53 | CYCLOPHOSPHAMIDE |
| TP53 | MENADIONE |
| TP53 | CHEMBL195350 |
| TP53 | CLEMASTINE |
| TP53 | ALPELISIB |
| TP53 | NORTRIPTYLINE |
| TP53 | CHEMBL374632 |
| TP53 | CHEMBL407501 |
| TP53 | 3R14S-OCHRATOXIN A |
| TP53 | TRAMETINIB |
| TP53 | METHYLBENZETHONIUM CHLORIDE |
| TP53 | THIORIDAZINE HYDROCHLORIDE |
| TP53 | TRANILAST |
| TP53 | INOSITOL |
| TP53 | CHLORFENAPYR |
| TP53 | DIBENZ[A,H]ANTHRACENE |
| TP53 | AZACITIDINE |
| TP53 | CERALASERTIB |
| TP53 | DASATINIB |
| TP53 | BAICALEIN |
| TP53 | VESNARINONE |
| TP53 | PERPHENAZINE |
| TP53 | TRIFLUOPERAZINE |
| TP53 | CHEMBL599088 |
| TP53 | GLUTARAL |
| TP53 | CHEMBL582715 |
| TP53 | CHEMBL546170 |
| TP53 | CHEMBL1303948 |
| TP53 | CHEMBL1494650 |
| TP53 | ASTEMIZOLE |
| TP53 | SELUMETINIB |
| TP53 | BENZO[B]FLUORANTHENE |
| TP53 | DICHLOROPHEN |
| TP53 | CHEMBL225513 |
| TP53 | PENTACHLOROPHENOL |
| TP53 | ADAVOSERTIB |

| | |
|---|---|
| TP53 | CLADRIBINE |
| TP53 | IFOSFAMIDE |
| TP53 | CHEMBL429335 |
| TP53 | TAMOXIFEN |
| RYR2 | SIMVASTATIN |
| RYR2 | DANTROLENE |
| RYR2 | CERIVASTATIN |
| RYR2 | ATORVASTATIN |
